# From surveillance to intelligence: a scoping review of machine learning for antimicrobial resistance surveillance intelligence across One Health

**DOI:** 10.3389/fpubh.2026.1922265

**Published:** 2026-09-17

**Authors:** Syed Arman Rabbani, Mohamed El-Tanani, Ismail I. Matalka, Shrestha Sharma, Manita Saini, Rakesh Kumar

**Affiliations:** 1 RAK College of Pharmacy, RAK Medical and Health Sciences University, Ras Al Khaimah, United Arab Emirates; 2 RAK Medical and Health Sciences University, Ras Al Khaimah, United Arab Emirates; 3 Amity Institute of Pharmacy, Amity University, Gurugram, Haryana, India; 4 Geeta Institute of Pharmacy, Geeta University, Panipat, Haryana, India; 5 Department of Pharmacy, Jagannath University, Bahadurgarh, Haryana, India

**Keywords:** antimicrobial resistance, artificial intelligence, early warning, forecasting, genomic surveillance, machine learning, One Health, public health decision support

## Abstract

**Background:**

Antimicrobial resistance (AMR) is a leading global health threat requiring coordinated surveillance across human, animal, environmental, and genomic systems. Machine learning is increasingly applied to AMR data, yet its contribution to actionable surveillance intelligence, rather than prediction alone, remains poorly defined.

**Objective:**

To map how machine-learning approaches generate AMR surveillance intelligence, to characterise their validation and implementation maturity, and to propose a framework distinguishing technical prediction from actionable surveillance intelligence.

**Methods:**

We conducted a scoping review following JBI methodology and PRISMA-ScR reporting. PubMed/MEDLINE, Scopus, and Web of Science were searched from January 2015 to May 2026 for studies applying machine learning or related methods to AMR surveillance intelligence. Two reviewers independently screened and charted records. Of 1,985 records, 66 met eligibility and formed the working evidence base; 41 studies (40 core empirical and one supporting preprint) were appraised against TRIPOD+AI- and PROBAST-aligned reporting, validation, and implementation-readiness domains.

**Results:**

Machine learning was applied across five clusters: clinical and electronic-health-record risk prediction and decision support; genomic and whole-genome-sequencing prediction; MALDI-TOF-based rapid resistance prediction; wastewater and metagenomic surveillance; and environmental, animal, food-chain, and One Health early warning. Prediction and risk stratification predominated, but validation maturity was limited: most studies were retrospective or internally validated, with few using external, cross-country, temporal, prospective, or drift-focused evaluation. On appraisal, discrimination was reported in 31 of 41 studies (76%) and explainability in 26 (63%); by contrast, external or temporal validation was present in only 15 (37%), calibration in 5 (12%), prospective evaluation in 1 (2%), and operational deployment with measured clinical or public-health impact in a single study (2%).

**Conclusion:**

Machine learning can support AMR surveillance intelligence across clinical, genomic, diagnostic, environmental, and One Health settings, but the evidence demonstrates technical feasibility far more convincingly than operational readiness. Realising this transition will require external and prospective validation, calibration and drift monitoring, transparent and equitable reporting, workflow integration, and explicit linkage of model outputs to clinical and public-health action. We propose a One Health AMR Surveillance Intelligence Framework to organise this shift from data generation toward actionable, adaptive surveillance intelligence.

## Graphical abstract

**Figure:**
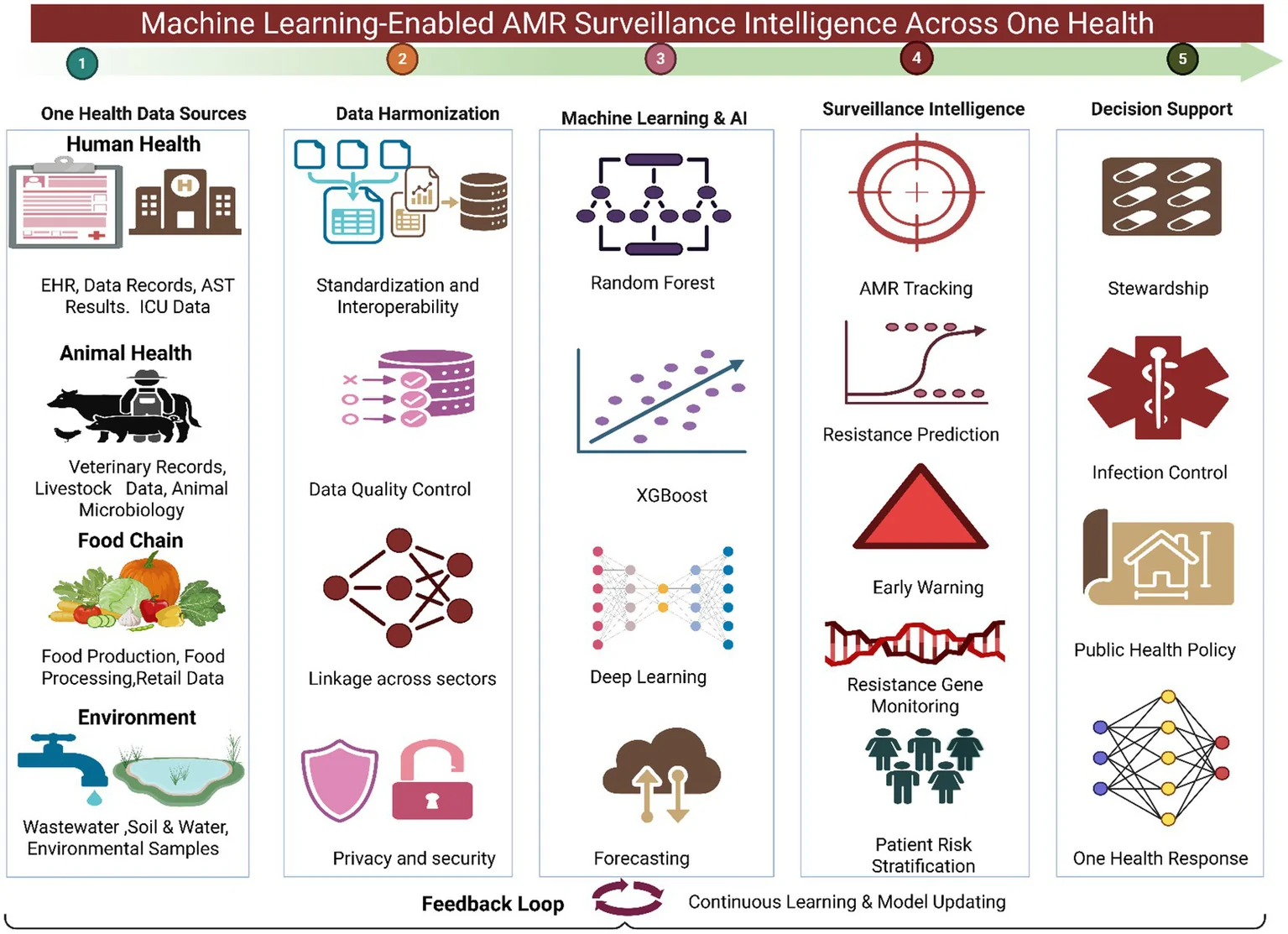
Machine learning-enabled AMR surveillance intelligence across One Health. One Health data sources (human, animal, food-chain, and environmental) are harmonised and analysed with machine-learning methods to generate surveillance-intelligence outputs, including AMR tracking, resistance prediction, early warning, resistance-gene monitoring, and patient risk stratification. These outputs drive decision support (stewardship, infection control, public-health policy, and One Health response), with a feedback loop for continuous learning and model updating.

## Introduction

Antimicrobial resistance (AMR) is among the most pressing global public-health threats. Bacterial AMR was directly responsible for an estimated 1.27 million deaths globally in 2019 and contributed to 4.95 million deaths in the same year (1, 2). Global Burden of Disease modelling attributes 1.14 million deaths to bacterial AMR in 2021, with 4.71 million associated deaths (3). Without effective intervention, AMR is projected to be directly responsible for 1.91 million deaths and associated with 8.22 million each year by 2050 (3).

AMR is not confined to hospitals or human medicine. It is a One Health problem (Figure 1) because resistant organisms, resistance genes, antimicrobial use, and environmental contamination can involve human, animal, food-production, plant, and ecosystem interfaces (1, 4). The Quadripartite One Health Joint Plan of Action frames health risks at the human-animal–plant-environment interface as requiring coordinated One Health action (4). For AMR, this means that surveillance systems must increasingly account for clinical infections, antimicrobial use, laboratory susceptibility data, animal and food-chain reservoirs, wastewater, environmental signals, genomic data, and population-level risk patterns, rather than relying on isolated laboratory reporting (1, 4, 5).

**Figure 1:**
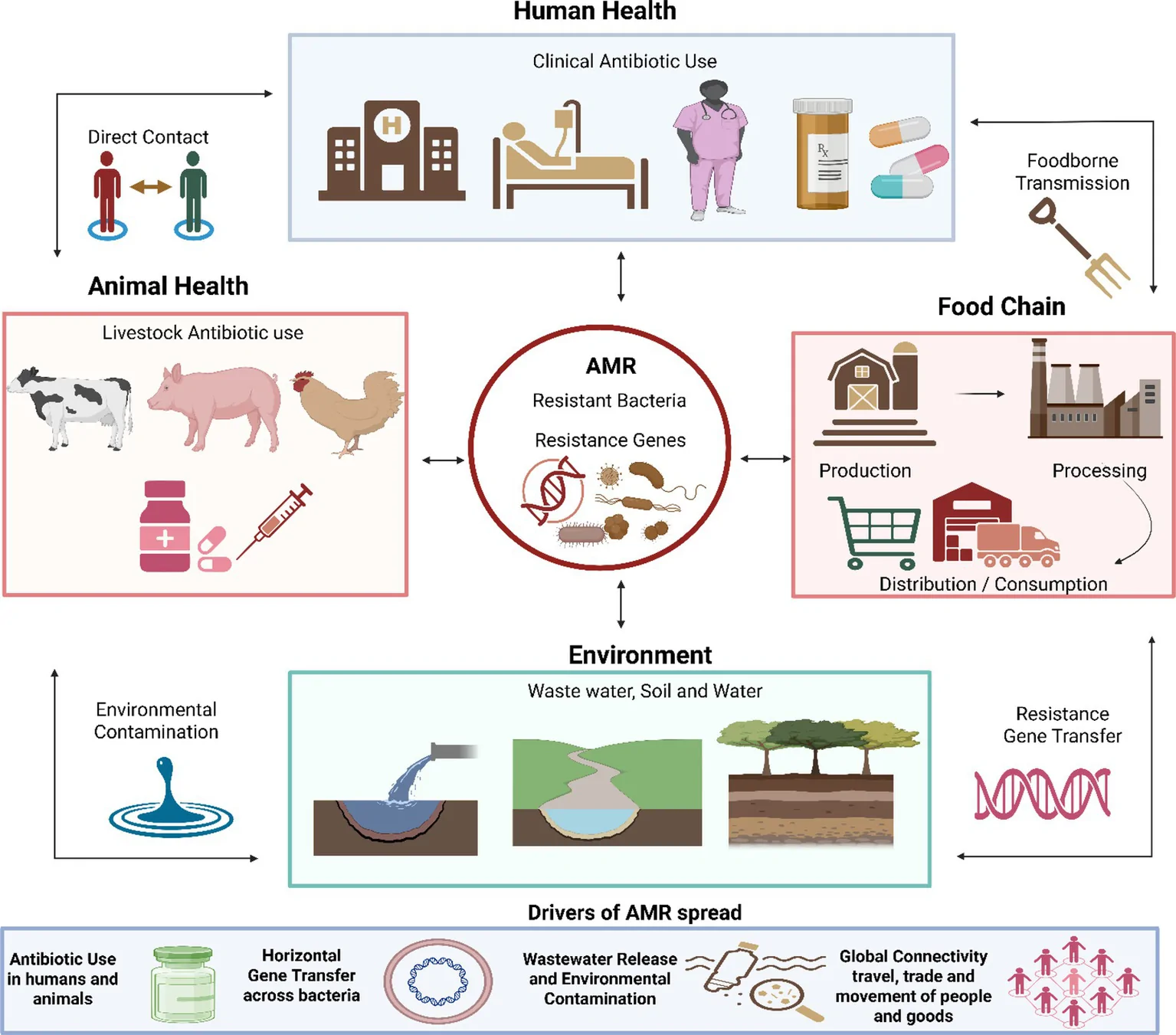
Antimicrobial resistance as a One Health problem: Transmission across sectors and drivers of spread. Resistant bacteria and resistance genes circulate among human health (clinical antibiotic use), animal health (livestock antibiotic use), the food chain (production, processing, distribution and consumption), and the environment (wastewater, soil and water) through direct contact, foodborne transmission, environmental contamination, and resistance-gene transfer. Major drivers of AMR spread include antibiotic use in humans and animals, horizontal gene transfer between bacteria, wastewater release and environmental contamination, and global connectivity (travel, trade, and movement of people and goods).

The global policy architecture has continued to move toward this integrated view. The 2024 WHO Bacterial Priority Pathogens List reprioritised 24 resistant pathogens across 15 families to direct research, development, and surveillance (6). In 2026, the World Health Assembly adopted a Global Action Plan on Antimicrobial Resistance for 2026 to 2036 that reaffirms coordinated One Health surveillance and response (7). At its 2024 high-level meeting, the United Nations General Assembly adopted a political declaration on antimicrobial resistance that set a target of reducing AMR-associated human deaths by 10% by 2030 (8).

Surveillance data alone do not automatically become actionable intelligence. Descriptive AMR surveillance can identify resistance patterns and monitor trends, but clinical and public-health decision-making increasingly requires earlier detection, prediction of future risk, recognition of high-risk patients or settings, identification of emerging resistance signals, and translation of those signals into timely interventions. The methodological question is therefore not only whether AMR can be predicted, but whether heterogeneous AMR-related data can be transformed into interpretable, validated, and decision-relevant intelligence for stewardship, infection prevention, laboratory diagnostics, environmental monitoring, and One Health response.

Machine learning (ML) and artificial intelligence (AI) have emerged as tools for this task, learning from high-dimensional clinical, microbiological, genomic, metagenomic, laboratory, and environmental data to support prediction, classification, forecasting, anomaly detection, and decision support (9–11). Prior reviews, however, have largely framed the field as a prediction problem. A 2022 systematic review and meta-analysis found that machine-learning approaches showed promise for AMR prediction but were constrained by retrospective development, non-standard data processing, and scarce real-world validation (9). A 2025 review of critical and high-priority pathogens reached similar conclusions, noting that gradient boosting, random forest, and XGBoost were among the better-performing model families (10). A translational review identified genomic prediction, mechanistic or drug-response discovery, and electronic-health-record-based stewardship as the principal application areas (11).

These reviews establish that machine learning can predict resistance, but prediction is only one component of surveillance intelligence. A model becomes useful for surveillance only when its outputs are timely, interpretable, validated in the target setting, and connected to a clinical, laboratory, public-health, environmental, or One Health decision pathway. Such a system can indicate where resistance is emerging, who or what is at risk, which sectors are involved, and whether interventions modify future risk.

A rapidly expanding literature now applies these methods across the full breadth of AMR-relevant data. Clinical, electronic-health-record, and laboratory information systems support patient-level prediction, risk stratification, and early susceptibility forecasting (12–24). Whole-genome and metagenomic sequencing support genotype-to-phenotype prediction and resistance-gene mapping (25–30). MALDI-TOF (matrix-assisted laser desorption/ionization time-of-flight) mass spectrometry enables rapid resistance inference (31–36). Wastewater, environmental, animal, and food-chain monitoring support population-level tracking, forecasting, and early warning (37–45).

Despite this breadth, the literature remains uneven in the dimensions that determine real-world usefulness, including external validation, temporal stability, prospective evaluation, calibration, and explicit linkage of model outputs to decisions. It has rarely been synthesised through a surveillance-intelligence lens that separates technical prediction from actionable, deployable intelligence.

This scoping review addresses that gap by mapping how machine learning and data-analytic approaches are being used to generate AMR surveillance intelligence across human, animal, environmental, food-chain, wastewater, genomic, diagnostic, and One Health contexts. Specifically, the review examines the data sources used, machine-learning methods applied, surveillance-intelligence functions supported, validation and implementation maturity, explainability, One Health integration, and remaining evidence gaps. The review also proposes a One Health AMR Surveillance Intelligence Framework to distinguish technical AMR prediction from actionable surveillance intelligence and to organise the transition from data generation toward risk stratification, forecasting, early warning, decision support, and learning feedback.

## Research gap and novel contribution

Prior reviews have advanced the field but have largely treated machine learning for AMR as a prediction task. Existing syntheses have summarised model families and reported performance for resistance prediction, yet they have given limited attention to whether model outputs are validated in the target setting, calibrated and monitored over time, interpretable, and connected to a defined clinical, laboratory, environmental, or One Health action (9–11). Three gaps follow from this. First, the literature has rarely been read through a surveillance-intelligence lens that separates technical prediction from validated, deployable intelligence. Second, evidence from clinical, genomic, diagnostic, environmental, wastewater, animal, and food-chain streams has usually been reviewed in isolation rather than mapped together across the One Health system. Third, the dimensions that determine operational usefulness, namely external and temporal validation, calibration, prospective evaluation, and explicit linkage to decisions, have not been charted systematically across these streams.

This scoping review addresses these gaps in three ways. It maps machine-learning evidence across the full One Health AMR data ecosystem within a single synthesis. It appraises each study against validation and implementation-readiness domains aligned with TRIPOD+AI and PROBAST rather than by performance alone. It then organises the findings into a One Health AMR Surveillance Intelligence Framework that distinguishes AMR data generation from actionable surveillance intelligence and identifies what is required to move from retrospective prediction toward validated and deployable use. The contribution is integrative and evaluative rather than a claim of primacy, and it is intended to reframe how the evidence is read and to guide the next stage of development.

## Methods

### Study design

We conducted a scoping review of machine learning-enabled AMR surveillance intelligence. A scoping-review design was selected because the evidence spans heterogeneous data streams, AMR outcomes, host sectors, modelling tasks, validation designs, and implementation stages. The review was informed by JBI scoping-review methodology and PRISMA-ScR reporting guidance. It was designed to map the evidence, identify conceptual and implementation gaps, and develop a synthesis framework rather than to estimate pooled model performance. The review protocol was registered on the Open Science Framework (OSF; registration https://doi.org/10.17605/OSF. IO/7WEM3). This article is a scoping review, and the conceptual framework presented in this work is a synthesis product of that review.

### Review question

The primary review question was: How are machine learning and data-analytic approaches being used to generate surveillance intelligence for AMR tracking, risk stratification, forecasting, early warning, and decision support across human, animal, environmental, and One Health systems? For this review, AMR surveillance intelligence was defined as the transformation of AMR-related data into timely, interpretable, and actionable information that can support clinical, laboratory, public-health, environmental, or One Health decision-making.

### Eligibility criteria

Eligibility criteria were structured using a population-concept-context framework.

The population or problem of interest was antimicrobial resistance in human, animal, environmental, food-chain, wastewater, genomic, metagenomic, laboratory, or One Health contexts. Studies were eligible if they addressed AMR, antimicrobial susceptibility or resistance prediction, multidrug-resistant organisms, resistance genes, resistome profiles, or pathogen-specific resistance outcomes.

The concept of interest was the use of machine learning, artificial intelligence, statistical learning, predictive modelling, forecasting, risk stratification, anomaly detection, genomic prediction, metagenomic analytics, MALDI-TOF spectral modelling, or related data-analytic approaches. Eligible studies had to include an analytic method that went beyond descriptive prevalence reporting.

The context of interest was surveillance intelligence. Eligible studies had to contribute to at least one surveillance-intelligence function. These functions were AMR tracking, patient-level risk prediction, antimicrobial susceptibility or resistance prediction, resistance prevalence forecasting, early warning, and hotspot mapping. They also included genomic, environmental or wastewater, and One Health surveillance, as well as laboratory decision support, antimicrobial stewardship decision support, and public-health response planning.

Studies were eligible if they were peer-reviewed original research articles, systematic reviews, scoping reviews, methodological papers, or framework papers directly relevant to AMR surveillance intelligence. The main empirical synthesis prioritised original modelling, surveillance, genomic, environmental, diagnostic, or One Health studies. Reviews and framework papers were retained as contextual evidence but were not counted as primary empirical evidence.

Studies were excluded if they focused only on antimicrobial drug discovery, molecular mechanisms of resistance without a surveillance or prediction component, general infectious-disease artificial intelligence without AMR relevance, descriptive AMR prevalence without analytic modelling, or non-AMR stewardship interventions. They were also excluded if they were opinion pieces without a methods or framework contribution, case reports, or conference abstracts without sufficient methodological detail, or if they could not be verified as eligible after full-text and source checking. The date range was January 2015 to May 2026. The language restriction was English-language publications.

### Information sources and search strategy

Three electronic bibliographic databases were searched: PubMed/MEDLINE, Scopus, and Web of Science. The search combined controlled-vocabulary terms, where available, with free-text keywords across three concept blocks. The first block covered antimicrobial resistance, for example antimicrobial or antibiotic resistance, multidrug-resistant organisms, resistance genes, resistome, and antimicrobial susceptibility. The second covered machine-learning and data-analytic methods, for example machine learning, artificial intelligence, deep learning, predictive modelling, forecasting, anomaly detection, genomic or metagenomic prediction, and MALDI-TOF spectral modelling. The third covered surveillance-intelligence functions, for example surveillance, tracking, risk stratification, forecasting, early warning, decision support, and One Health. The three concept blocks were combined with Boolean operators. Within each block, controlled-vocabulary terms and free-text keywords were combined with OR, and the three concept blocks were combined with AND. Controlled vocabulary comprised MeSH headings in PubMed/MEDLINE and the equivalent index or topic fields in Scopus and Web of Science, with free-text terms searched in title, abstract, and keyword fields. Records from all databases were exported to reference-management software, where duplicates were removed automatically and then checked manually before screening. Searches were restricted to English-language records published from January 2015 onward, and all databases were last searched on 1 May 2026. Database searching was supplemented by backward and forward citation chasing. The full database-specific search strategies, including all search terms, field tags, and limits, are provided in Supplementary File S1.

### Record management and study selection

Search results from each database were imported into reference-management software and de-duplicated, with manual checking of uncertain matches. Screening was conducted in two stages by two reviewers working independently, with disagreements resolved by discussion and consensus. First, titles and abstracts were screened against the eligibility criteria. Records were classified as include, exclude, maybe or uncertain, or background/contextual. Records were advanced to full-text screening if they clearly met the eligibility criteria or if relevance could not be confidently resolved from the title and abstract. At full-text screening, reports were excluded for mutually exclusive primary reasons, which are summarised with their counts in the PRISMA-ScR flow diagram (Figure 2). The number of records identified, screened, assessed at full text, and excluded with reasons is reported in the Results and in Figure 2.

**Figure 2:**
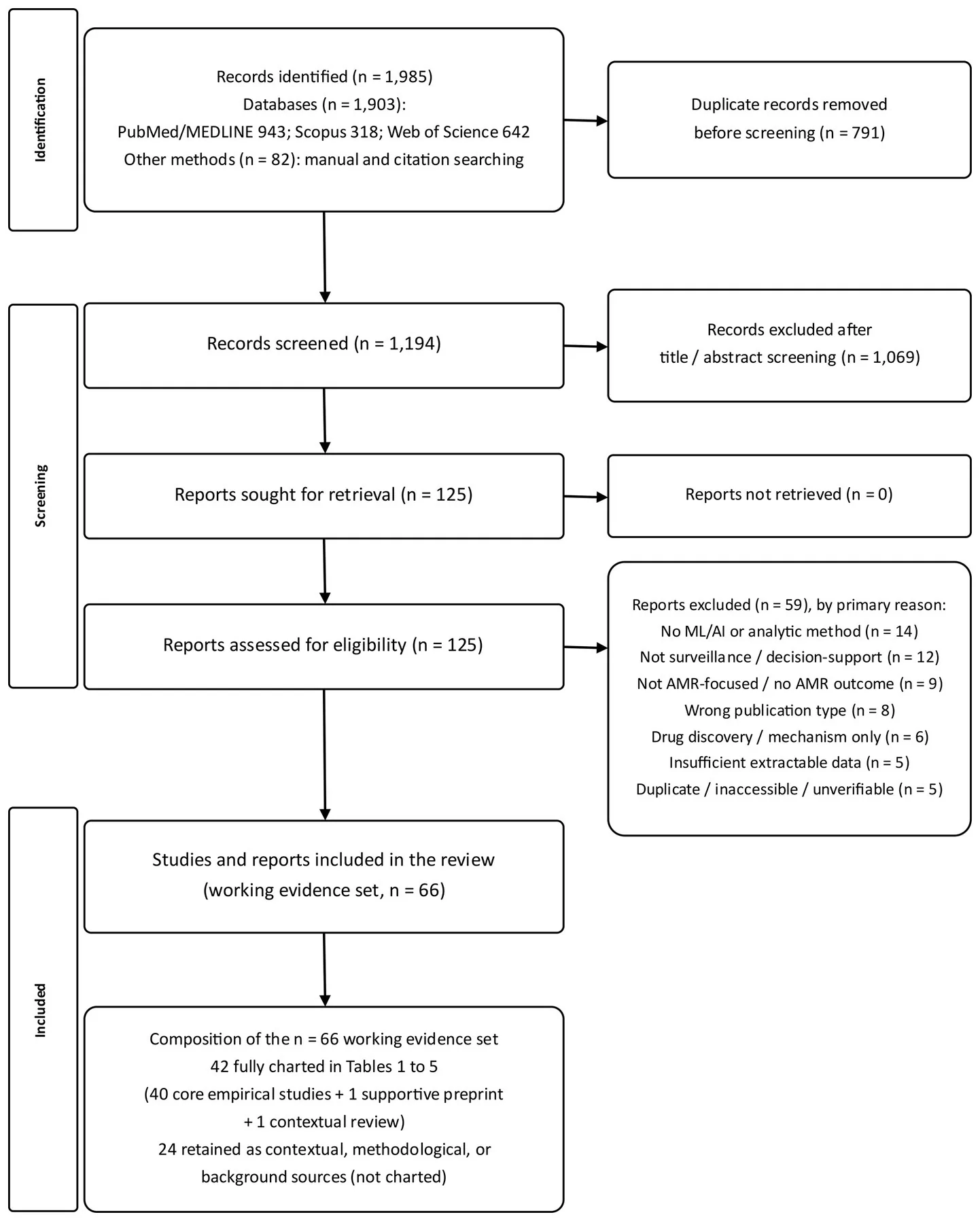
PRISMA-ScR flow diagram for review.

### Data extraction

Data were charted independently by two reviewers into a structured review-specific data collection form. The extraction framework included bibliographic information, study design, country or region, AMR domain, pathogen or organism, data source, sample size or dataset size where reported, AMR outcome, machine-learning or analytic method, surveillance-intelligence function, validation design, performance metrics, explainability approach, implementation readiness, One Health relevance, limitations, evidence role, and source-verification status. The main surveillance-intelligence functions coded were AMR tracking, AMR or antimicrobial susceptibility prediction, patient-level risk stratification, resistance prevalence forecasting, early warning, hotspot mapping, laboratory or clinical decision support, One Health integration, and framework or surveillance-system development.

### Quality, validation, and implementation appraisal

Because this was a scoping review across heterogeneous study types, studies were not excluded solely on the basis of formal risk-of-bias appraisal. Instead, each study was assessed using structured quality, validation, and implementation-readiness domains relevant to AMR surveillance intelligence. Domains included data source and data quality, AMR outcome definition, model development approach, validation design, performance reporting, calibration or threshold reporting where available, explainability, external validity and transportability, implementation readiness, One Health integration, and source-verification caveats.

Each study was additionally appraised against the reporting items of TRIPOD+AI (46) and the risk-of-bias and applicability concepts of PROBAST and its artificial-intelligence update PROBAST+AI (47, 48). Scoring used only reported information across validation design, external or temporal validation, calibration, decision-analytic utility, prospective evaluation, explainability, and operational deployment.

### Evidence synthesis

A structured narrative synthesis was conducted. Studies were grouped by data source, AMR domain, machine-learning method, surveillance-intelligence function, validation maturity, implementation readiness, and One Health relevance. Quantitative pooling was not performed because the studies differed substantially by pathogen, AMR endpoint, data source, model architecture, validation design, and performance metric. Counts are therefore reported as synthesis-set counts and should not be interpreted as prevalence estimates for the entire AMR machine-learning literature. The review was reported in line with the PRISMA-ScR checklist (49). The completed PRISMA-ScR checklist is provided as Supplementary File S2.

### Development of the conceptual framework

A conceptual framework was developed from the structured synthesis. The framework was designed to distinguish AMR data generation from actionable surveillance intelligence. Evidence from clinical, laboratory, genomic, environmental, wastewater, food-chain, animal-sector, and One Health studies was mapped onto six functional layers: One Health data streams, data harmonization and governance, machine-learning and analytics engine, surveillance-intelligence outputs, decision support and public-health action, and a learning feedback loop. The framework was not intended to imply that all included studies had operational deployment or prospective evaluation. Rather, it was used to organise the evidence and identify requirements for moving from retrospective AMR prediction toward validated, interpretable, and actionable surveillance intelligence.

## Results

### Study selection

The searches identified 1,985 records before deduplication: 943 from PubMed/MEDLINE, 318 from Scopus, 642 from Web of Science, and 82 from manual or supplementary screening. After removing 791 duplicates, 1,194 records were screened by title and abstract; 1,069 were excluded; 125 full-text reports were assessed; and 59 were excluded. The full-text exclusions were assigned to mutually exclusive primary reasons: no ML/AI or comparable analytic method (*n* = 14), not surveillance-intelligence or decision-support relevant (*n* = 12), not AMR-focused or no AMR/resistance outcome (*n* = 9), wrong publication type for empirical synthesis (*n* = 8), drug discovery/basic mechanism focus without surveillance relevance (*n* = 6), insufficient extractable data (*n* = 5), and duplicate/overlapping reports, inaccessible full texts, or insufficient source verification (*n* = 5). Sixty-six studies/reports were retained in the working evidence set. The data-extraction set comprised 42 sources, and the core empirical synthesis set comprised 40 studies. The remaining 24 were retained as contextual, methodological, or background sources that were not charted. Figure 2 shows the PRISMA-ScR flow.

### Evidence set used for synthesis

The final working evidence set retained after full-text assessment comprised 66 studies or reports. The synthesis tables focus on the 42 sources that underwent full data extraction. Forty studies were classified as core empirical evidence, one was retained as supportive preprint evidence, and one was contextual review/framework evidence. The remaining 24 sources were retained as contextual, methodological, reporting-standard, or framework sources that informed interpretation but were not charted as study-level empirical evidence.

### Study characteristics

The evidence base was dominated by original empirical or modelling studies. Only one of the 42 data-extraction sources was a systematic review, Ardila et al., which was retained for contextual interpretation rather than as primary empirical evidence (10). The core empirical studies covered hospital and intensive-care risk prediction, large-scale surveillance database modelling, genomic and metagenomic AMR prediction, wastewater and environmental surveillance, food-chain or animal-sector surveillance, One Health surveillance, and MALDI-TOF-based rapid resistance prediction. Table 1 presents the characteristics of the included studies.

**Table 1:** Characteristics of the included studies.

Studies	Study type	Role in synthesis	Domain/setting	References
Ardila et al., 2025	Systematic review	Contextual review/framework evidence	Real-world healthcare AST studies	(10)
Ferrari et al., 2024	Original empirical study	Core empirical evidence	ICU EHR-based early alert/antimicrobial stewardship	(12)
Diaz et al., 2025	Original empirical study	Core empirical evidence	Clinical/EHR-based risk prediction	(13)
Ureña et al., 2024	Original empirical study	Core empirical evidence	Hospital laboratory information system	(14)
Hardan et al., 2024	Original study	Core empirical evidence	MIMIC-IV multimodal EHR	(15)
Hendriksen et al., 2019	Original study	Core empirical evidence	Urban sewage metagenomic surveillance	(37)
Weis et al., 2022	Original study	Core empirical evidence	Clinical MALDI-TOF/diagnostics	(31)
Ren et al., 2024	Original study	Core empirical evidence	MALDI-TOF nosocomial pathogen screening	(32)
Vihta et al., 2024	Original empirical study	Core empirical evidence	Hospital Trusts/national hospital network	(16)
Valavarasu et al., 2025	Original empirical study	Core empirical evidence	Global surveillance database analysis	(17)
Nsubuga et al., 2024	Original empirical study	Core empirical evidence	Genomic AMR prediction and cross-country validation	(25)
Feng et al., 2025	Original empirical study	Core empirical evidence	Regional One Health resistome surveillance	(38)
Feng B et al., 2025	Original empirical study	Core empirical evidence	Environmental ARG pollution and health-risk prediction (rural domestic sewage)	(39)
Yu et al., 2026	Original empirical study	Core empirical evidence	Clinical early risk stratification before final AST results	(18)
Mutahwa et al., 2025	Preprint/original empirical study	Supportive preprint evidence	Provincial hospital AMR surveillance in resource-limited setting	(19)
Zhao et al., 2025	Original empirical study	Core empirical evidence	ICU MDRO risk prediction/clinical decision support	(20)
Duroux et al., 2026	Original empirical study	Core empirical evidence	Rapid AMR prediction across hospitals and unseen time periods	(33)
Li et al., 2024	Original study	Core empirical evidence	ICU admission/EHR	(21)
Lewin-Epstein et al., 2021	Original study	Core empirical evidence	Hospitalised patients/EMR	(22)
Nigo et al., 2024	Original study	Core empirical evidence	Hospital EHR/time-series prediction	(23)
Haredasht et al., 2025	Conference proceedings/original study	Core empirical evidence	Inpatient EHR/stewardship	(24)
Woh et al., 2025	Original study	Core empirical evidence	Foodborne pathogen genomic surveillance	(26)
Van Camp et al., 2020	Original study/tool	Core empirical evidence	WGS-based clinical decision support tool	(27)
Gao et al., 2024	Original study	Core empirical evidence	WGS rapid AMR prediction	(28)
Pan et al., 2025	Original study	Core empirical evidence	WGS and mNGS rapid AST prediction	(29)
Zhang et al., 2022	Original study	Core empirical evidence	Global metagenomic ARG risk mapping	(30)
Secker et al., 2026	Original study	Core empirical evidence	Urban–rural wastewater metagenomics	(40)
Knight et al., 2024	Original study	Core empirical evidence	National wastewater surveillance	(41)
López-Cortés et al., 2025	Original study	Core empirical evidence	VITEKMS MALDI-TOF clinical spectra	(34)
Feucherolles et al., 2022	Original study	Core empirical evidence	Foodborne pathogen MALDI-TOF AMR screening	(35)
Wiesmann et al., 2025	Original validation study	Core empirical evidence	Routine diagnostics MALDI-TOF validation	(36)
Jeffrey et al., 2020	Original modelling study	Core empirical evidence	National/regional surveillance forecasting	(42)
Csorba 2025a	Original modelling study	Core empirical evidence	Environmental AMR risk early warning	(43)
Csorba 2025b	Original modelling study	Core empirical evidence	Broiler production chain	(44)
Kim et al., 2023	Original observational/modelling study	Core empirical evidence	Farm-level AMR surveillance	(45)
Martínez-Agüero et al., 2022	Original modelling study	Core empirical evidence	ICU clinical time-series prediction	(50)
Kanjilal et al., 2020	Retrospective cohort; ML decision algorithm	Core empirical evidence	Outpatient uncomplicated UTI, USA	(51)
Shapiro Ben David et al., 2025	Real-world deployment/implementation study	Core empirical evidence	Outpatient UTI, Israel	(52)
Nguyen et al., 2019	Retrospective ML genomic modelling	Core empirical evidence	Foodborne *Salmonella* surveillance, USA	(53)
Yagimoto et al., 2024	Retrospective ML method study	Core empirical evidence	Genomic ARG mechanism prediction	(54)
Munk et al., 2022	Metagenomic surveillance study	Core empirical evidence	Global wastewater/One Health	(55)
Oonsivilai et al., 2018	Retrospective ML modelling	Core empirical evidence	Paediatric sepsis, Cambodia (LMIC)	(60)

The clinical studies used electronic health records (EHRs), microbiology laboratory data, laboratory information systems (LIS), antimicrobial susceptibility testing (AST) results, complete blood count (CBC) variables, or intensive-care unit (ICU) time-series data. Examples include ICU EHR-based early alert prediction, blood-culture AMR prediction using EHR and socioeconomic deprivation indices, and early forecasting of antimicrobial susceptibility from bacteriology workflow data (12–14). Other examples include multimodal EHR prediction, carbapenem-resistant *Pseudomonas aeruginosa* (CRPA) risk stratification, and ICU multidrug-resistant organism (MDRO) risk prediction (15, 18, 20). Additional examples include electronic medical record (EMR)-based resistance prediction, methicillin-resistant *Staphylococcus aureus* (MRSA) culture-positivity prediction, and interpretable ICU time-series prediction of multidrug resistance (22, 23, 50).

### Data sources and AMR targets

The reviewed studies showed that AMR surveillance intelligence is being developed from multiple data streams rather than from a single dominant data type (Table 2). Clinical and hospital studies used EHRs, EMRs, ICU records, microbiology data, AST results, and laboratory workflow data. Genomic studies used whole-genome sequencing (WGS) or metagenomic sequencing data to predict AMR phenotypes, identify resistance markers, or map resistance-gene risk. MALDI-TOF studies repurposed routine diagnostic mass-spectrometry spectra for rapid AMR prediction or screening. Environmental and wastewater studies used sewage, wastewater, environmental indicators, metagenomic resistome profiles, quantitative PCR (qPCR), agricultural indicators, and geospatial variables.

**Table 2:** Data sources, AMR domains, and outcomes.

Studies	AMR domain	Country/region	Data source/sample	Pathogen/target	Resistance outcome	References
Ardila et al., 2025	Human	Global	Hospital information systems and antimicrobial susceptibility tests; 21 included papers; 688,107 patients; 1,710,867 AST results	Critical and high-priority pathogens	AMR prediction using AST	(10)
Ferrari et al., 2024	Human	United Kingdom	EHR/intensive care, microbiology, antimicrobial treatment, intervention, bed occupancy/staffing data at ICU admission; Initial >5,000 ICU patients; final complete-case dataset 1,142 patients	Bloodstream infection and AMR in ICU patients	Presence of BSI and AMR	(12)
Diaz et al., 2025	Human	United States	79,529 blood cultures from 58,017 unique individuals; EHR demographics, prior infection/resistance, comorbidities, insurance, ADI/SVI socioeconomic indices, microbiology/susceptibility data.	ESBL, CRE, AmpC, MRSA, VRE	AMR organisms in blood cultures	(13)
Ureña et al., 2024	Human	France	44,026 antibiogram instances over 7 years; cleaned dataset of 30,975 antibiograms from 13,166 patients; 26,621 antibiograms from 2014–2019 for training/testing and 4,354 from 2020 for validation.	Hospitalised patient bacterial pathogens	Antimicrobial susceptibility probability	(14)
Hardan et al., 2024	Human health	USA/MIMIC-IV	MIMIC-IV ICU EHR database; modalities: time-invariant data, time-series clinical/lab/microbiology/procedure data and clinical notes; Gentamicin task used ~1,245–1,322 ICU stays depending on prediction time; *P. aeruginosa* dataset also constructed.	AMR prediction by antibiotic or pathogen	Resistance prediction	(15)
Hendriksen et al., 2019	Environmental/global	60 countries	Untreated sewage from 79 sites in 60 countries; >1.4 Tb sequence data; average 120 million reads/sample; World Bank health/development variables used for predictive modelling.	AMR genes/resistome	AMR gene abundance and diversity	(37)
Weis et al., 2022	Human health/rapid diagnostics	Switzerland	DRIAMS-A-D routine diagnostic MALDI-TOF dataset; 303,195 mass spectra and 768,300 AMR labels from 803 bacterial/fungal species; main DRIAMS-A analysis included 145,341 spectra.	*S. aureus*, *E. coli*, *K. pneumoniae* and other clinical isolates	AMR phenotype prediction from MALDI-TOF spectra	(31)
Ren et al., 2024	Human health	Switzerland/DRIAMS diagnostic laboratory data	DRIAMS mass-spectra dataset; DRIAMS-A primary training/development, DRIAMS-B–D external site testing; >4,000 samples for 19 of 20 antibiotics; teicoplanin had 488 samples.	*Staphylococcus epidermidis*	Antibiotic resistance prediction	(32)
Vihta et al., 2024	Human	England	UKHSA SGSS bloodstream infection surveillance linked to England NHS Trusts; 22 pathogen-antibiotic combinations; FY2016/17–FY2021/22	Bloodstream infection pathogen-antibiotic combinations	Future AMR prevalence	(16)
Valavarasu et al., 2025	Human	Global/Pfizer ATLAS	Pfizer ATLAS dataset; 917,049 bacterial isolates; phenotype-only and phenotype+genotype subsets; 589,998 genotype-linked isolates	Bacterial isolates across surveillance dataset	Antibiotic resistance outcome	(17)
Nsubuga et al., 2024	Human	England; Uganda; Nigeria; Tanzania	Models trained on England *E. coli* WGS and validated using independent African datasets from Uganda, Nigeria, and Tanzania; target antibiotics ciprofloxacin, ampicillin, cefotaxime	*E. coli*	AMR prediction from WGS	(25)
Feng et al., 2025	One Health	China	592 samples from humans, food, and environment in Dengfeng, China; metagenomic sequencing plus culture of resistant isolates	ARGs and carbapenem-resistant strains	ARG flow and carbapenem-resistant strains	(38)
Feng B et al., 2025	Environment	China (39 sites across 22 provinces)	Metagenomic sequencing of rural domestic sewage; 702 ARG subtypes across 21 ARG types; antibiotic and environmental covariates.	Antimicrobial resistance genes (ARGs); host bacteria *Klebsiella pneumoniae* and *Escherichia coli*	ARG pollution level and health-risk index (multidrug resistance predominant)	(39)
Yu et al., 2026	Human	China (primary and external validation hospital cohorts)	1,666 *P. aeruginosa*-infected patients in primary center plus external validation cohort *n* = 471; CBC and demographic variables	*Pseudomonas aeruginosa*/CRPA	Carbapenem-resistant *Pseudomonas aeruginosa* risk	(18)
Mutahwa et al., 2025	Human	Zimbabwe	Gweru Provincial Hospital, Zimbabwe; 4,054 cleaned clinical isolate records from 874 patient records, 2022–2024; 80/20 train-test split.	Clinical isolates across hospital records	Resistance prediction/AMR patterns	(19)
Zhao et al., 2025	Human	China	Retrospective cohort of ICU patients in China; 825 eligible cases, 375 MDRO infections	MDRO infections in ICU patients	MDRO infection risk	(20)
Duroux et al., 2026	Human	Switzerland/DRIAMS diagnostic laboratory network	DRIAMS data from 4 Swiss diagnostic laboratories; 54,283 spectra; 631,167 AMR phenotypes; 65 antimicrobials	AMR from MALDI-TOF spectra/antimicrobials	Rapid AMR prediction in clinical settings	(33)
Li et al., 2024	Human health	China; USA MIMIC-IV	PLAGH-ICU *n* = 3,536 and MIMIC-IV *n* = 34,923; variables within 24 h of ICU admission	MDRO colonisation or infection	MDRO colonisation/infection risk within 24 h ICU admission	(21)
Lewin-Epstein et al., 2021	Human health	Israel	Rabin Medical Center EMR and culture data, 2013–2015; five antibiotics: ceftazidime, gentamicin, imipenem, ofloxacin, sulfamethoxazole-trimethoprim	Bacterial infections in hospitalised patients	Antibiotic resistance profile	(22)
Nigo et al., 2024	Human health	USA	Memorial Hermann development data: 8,164 MRSA and 18,069 non-MRSA patients; external MIMIC-IV validation with 393,713 culture events	MRSA	Positive MRSA culture within two weeks	(23)
Haredasht et al., 2025	Human health	USA	Stanford STARR EHR data; 49,872 adult inpatient encounters with positive urine, blood, or respiratory cultures; training 2009–2021 (*n* = 38,312), temporal test 2022–2023 (*n* = 11,560).	Urine, blood, and respiratory infections	Resistance/susceptibility to cefazolin, ceftriaxone, cefepime, piperacillin-tazobactam, and ciprofloxacin	(24)
Woh et al., 2025	Food-chain/public health/genomic surveillance	Hong Kong and ASEAN countries	NCBI Pathogen Detection *Salmonella* WGS; 788 isolates with genotype and phenotype data; sources included food (*n* = 372), humans (*n* = 173), environment (*n* = 154), and animals (*n* = 89).	Nontyphoidal *Salmonella*; S. Weltevreden ST365 was the most common serovar/sequence type cluster.	Prediction of resistance across 16 antimicrobial agents from WGS-derived AMR gene features.	(26)
Van Camp et al., 2020	Human health	Not country-specific; public NCBI BioSample WGS data	NCBI BioSample WGS data for Gram-negative bacteria; prediction for eight antibiotics	Gram-negative bacteria	Drug resistance prediction	(27)
Gao et al., 2024	Human health	China	339 *A. baumannii* isolates for model construction; independent test dataset of 120 isolates; 13 antimicrobial agents	*Acinetobacter baumannii*	MIC/category prediction	(28)
Pan et al., 2025	Human health	China	1,054 ECC strain genomes with AST data from public databases and Shanghai Children’s Hospital, plus 104 paediatric respiratory specimens for mNGS-AST validation.	*Enterobacter cloacae* complex	Antibiotic resistance prediction	(29)
Zhang et al., 2022	Environmental/global One Health	Global habitats	4,572 metagenomic samples from six habitat types; 2,561 ARGs across 24 antibiotic classes	Antibiotic resistance genes	ARG health-risk scoring and global marine threat mapping	(30)
Secker et al., 2026	Environmental/wastewater	India	Nagpur, India; 422 grab samples pooled into 138 composite wastewater samples across 10 urban zones and rural catchments	ARGs and bacterial pathogens in untreated wastewater	ARG/pathogen reservoir mapping	(40)
Knight et al., 2024	Environmental/wastewater	Wales, UK	47 WWTPs across Wales; weekly composite influent samples; daily hospital effluent samples for 4 weeks	ARGs/resistome	AMR gene quantification and resistome profiling	(41)
López-Cortés et al., 2025	Human health	Chile	2,229 mass spectra from VITEK MS; *S. aureus*, *E. coli*, *K. pneumoniae* and other isolates from Regional Hospital of Talca	Clinical pathogens including *E. coli*, *K. pneumoniae*, *S. aureus*	Antibiotic resistance prediction	(34)
Feucherolles et al., 2022	Food-chain/One Health	Luxembourg/One Health panel	One Health *Campylobacter* panel: 224\u00B0*C. jejuni* and 116\u00B0*C. coli* strains; seven antimicrobial resistances tested	*Campylobacter* spp.	AMR screening/resistance phenotype	(35)
Wiesmann et al., 2025	Human health	Germany; Switzerland DRIAMS-A	Routine diagnostics from Germany plus DRIAMS-A; *E. coli n* = 7,897, *K. pneumoniae n* = 2,444, *S. aureus n* = 4,664; MALDI-TOF spectra linked to AST.	*S. aureus*; *E. coli*; *K. pneumoniae*	AMR prediction from MALDI-TOF spectra	(36)
Jeffrey et al., 2020	Human	Europe; England	EARS-Net annual country-level resistance data for Europe, 2012–2016; England Fingertips quarterly CCG *E. coli* resistance data, 2015Q4-2018Q4; linked antibiotic consumption/prescribing, bacteraemia, GDP, and ILI covariates.	Antibiotic resistance prevalence across countries/clinical commissioning groups	Antibiotic resistance prevalence	(42)
Csorba 2025a	Environment	Global/environmental indicators	High-level environmental/agricultural indicators including pesticide use, land-use change, precipitation, crop type, population density, fertilizer application	Potential AMR risk profiles	Anomalous environmental profiles associated with AMR risk	(43)
Csorba 2025b	Food-chain/animal health	International databases/poultry production	International high-level agricultural/environmental indicators focused on ciprofloxacin resistance in *Campylobacter* in broiler production	*Campylobacter*	Ciprofloxacin resistance	(44)
Kim et al., 2023	Animal health/food animal production	United States	Pathogen and antimicrobial data from >600 US farms, 2010–2021; five bacterial pathogens	Bacterial pathogens in food animal production	Future AMR burden	(45)
Martínez-Agüero et al., 2022	Human	Spain	ICU time-series EHR data from University Hospital of Fuenlabrada, Madrid, Spain	Multidrug-resistant bacteria in ICU patients	Early prediction of antimicrobial multidrug resistance	(50)
Kanjilal et al., 2020	Human	USA	EHR uncomplicated UTI specimens	Uropathogens including *E. coli*	Antibiotic susceptibility prediction	(51)
Shapiro Ben David et al., 2025	Human	Israel	EHR and laboratory data; 171,010 UTI episodes	Uropathogens	Predicted resistance; antibiotic mismatch	(52)
Nguyen et al., 2019	Human/food	USA	WGS; 5,278 nontyphoidal *Salmonella* genomes	Nontyphoidal *Salmonella*	MIC prediction for 15 antibiotics	(53)
Yagimoto et al., 2024	Molecular/genomic	Not country specific	ARG protein-sequence datasets	Multiple ARG families	Resistance-mechanism prediction	(54)
Munk et al., 2022	Environmental/One Health	101 countries	Sewage metagenomics; 757 samples, 243 cities	Resistome (ARGs)	Global ARG abundance and transmission	(55)
Oonsivilai et al., 2018	Human	Cambodia	Blood-culture and clinical data; 243 bloodstream infections	Bloodstream pathogens	Susceptibility to empiric regimens	(60)

AMR, antimicrobial resistance; AST, antimicrobial susceptibility testing; BSI, bloodstream infection; EHR, electronic health record; ICU, intensive care unit; LIS, laboratory information system; MALDI-TOF, matrix-assisted laser desorption/ionization time-of-flight mass spectrometry; WGS, whole-genome sequencing.

### Machine-learning and analytical approaches

Tree-based machine-learning methods were common across clinical, surveillance database, genomic, and environmental studies (Table 3). Random forest, XGBoost, LightGBM, gradient boosting, and CatBoost were used in several clinical, surveillance, genomic, and MALDI-TOF examples (13, 17, 34). Deep-learning approaches were used in multimodal EHR prediction, MRSA time-series prediction, and interpretable ICU time-series modelling (15, 23, 50). Bayesian, probabilistic, and time-series approaches appeared in early susceptibility forecasting, broiler-chain early warning, and farm-level AMR time-series prediction (14, 44, 45).

**Table 3:** Machine-learning methods and surveillance-intelligence functions.

Studies	ML/analytics method	Task type	Function/output	Explainability	Implementation readiness	References
Ardila et al., 2025	GBDT, Random Forest, XGBoost, Logistic Regression, Neural Networks and other ML approaches across included studies	Systematic review/prediction	Contextual evidence for AMR prediction in real-world healthcare settings	Not applicable	R1 Conceptual	(10)
Ferrari et al., 2024	Multi-Objective Symbolic Regression (MOSR), compared against LR, DT, RF, GB, XGBoost, LightGBM, LDA/QDA, Extra Trees, AdaBoost; HSIC-Lasso feature selection; SMOTE sensitivity/balancing scenario	Early alert prediction	Early alert/risk prediction for ICU BSI and AMR to support antimicrobial stewardship	Symbolic regression readable equations	R2 Retrospective model	(12)
Diaz et al., 2025	Penalised logistic regression, random forest, and XGBoost; class weighting for imbalance; 5-fold stratified grid-search tuning.	Risk classification	Patient-level risk stratification and empirical antibiotic decision support while awaiting culture/susceptibility results.	Not specified	R2 Retrospective model	(13)
Ureña et al., 2024	Bayesian models, neural networks, XGBoost, gradient boosting, AdaBoost, bagging, random forest, logistic regression and frequentist baseline models; SHAP for NN interpretability.	Early forecasting/prediction	Early forecasting of susceptibility before final antibiogram; staged laboratory-decision support for empiric therapy.	Explains contribution of cofactors	R2 Retrospective model	(14)
Hardan et al., 2024	ClinicalBERT, LSTM/StarTransformer/Transformer encoders; MAGBERT, tensor fusion, attention fusion and MultiModal Infomax (MMIM) fusion.	Classification/multimodal prediction	Real-time patient-level AMR prediction from routinely available EHR data; potential EHR workflow integration.	Not specified	R2 Retrospective model	(15)
Hendriksen et al., 2019	Metagenomic resistome profiling; ordination/clustering; generalised mixed models; predictive modelling using country-level health, sanitation and development indicators.	Global monitoring/prediction	Global AMR tracking, environmental surveillance, geographic prediction and population-level early-warning substrate.	Not specified	R2 Retrospective model	(37)
Weis et al., 2022	Logistic regression, LightGBM, and multilayer perceptron classifiers using 6,000-dimensional binned MALDI-TOF spectra; fivefold cross-validation hyperparameter search; SHAP feature-contribution analysis.	Rapid prediction/diagnostic decision support	Rapid diagnostic intelligence for earlier resistance inference and antimicrobial stewardship support.	SHAP values and feature-bin analysis	R3 External/temporal validation	(31)
Ren et al., 2024	RF meta-transformer feature selection; RF, logistic regression, Naïve Bayes, SVM, LightGBM and MLP; grid-search tuning; SHAP biomarker interpretation.	Rapid prediction/screening	Rapid MALDI-TOF-based resistance profiling and nosocomial infection decision support.	SHAP and protein biomarker analysis	R3 External/temporal validation	(32)
Vihta et al., 2024	XGBoost compared with last-value-carried-forward, difference-carried-forward, and linear trend forecasting; SHAP for interpretation	Forecasting	Directly supports T4 forecasting and T1 tracking at aggregate hospital/network level	SHAP feature importance	R3 External/temporal validation	(16)
Valavarasu et al., 2025	Multiple ML models; XGBoost best; hyperparameter tuning; balancing; SHAP	Classification/prediction	T2 prediction; surveillance-database analytics; decision-support/policy relevance	SHAP summary plots	R2 Retrospective model	(17)
Nsubuga et al., 2024	SVM, LightGBM, Gradient Boosting, Logistic Regression, Random Forest; mutation identification	Prediction/generalizability	T2 prediction; V5 cross-country validation; LMIC/equity relevance	Key mutations identified	R3 External/temporal validation	(25)
Feng et al., 2025	Metagenomic profiling; microbial/ARG flow analysis; ML models based on microbiome profiles for carbapenem-resistant strain prediction	Prediction/One Health transmission mapping	T8 One Health integration; T6 hotspot/transmission mapping; T2 prediction	Not specified	R2 Retrospective model	(38)
Feng B et al., 2025	Machine-learning risk-prediction models; partial least-squares path modelling and redundancy analysis for driver attribution.	Risk prediction	National-scale prediction of ARG health risk in rural domestic sewage and identification of antibiotic drivers; geographic risk mapping.	Driver attribution via partial least-squares path modelling and redundancy analysis	R2 Retrospective model	(39)
Yu et al., 2026	LASSO feature selection; eight ML algorithms; RF best; SHAP; DCA; Bayesian hyperparameter optimization; SMOTE sensitivity analysis	Risk stratification	T3 risk stratification; T7 decision support; V4 external validation	SHAP; decision curve analysis; calibration/Brier score	R3 External/temporal validation	(18)
Mutahwa et al., 2025	SVM, random forest, logistic regression, gradient boosting, and KNN; grid search with 10-fold stratified cross-validation and 80/20 holdout testing.	Predictive surveillance	T2 prediction; T1 tracking; LMIC relevance	Feature importance analysis	R2 Retrospective model	(19)
Zhao et al., 2025	Random Forest, neural networks, SVM, logistic regression, decision tree, Gaussian NB; LASSO; SHAP; DCA	Risk stratification	T3 risk stratification; T7 decision support; explainable AI	SHAP global/local explanations; dynamic force plots	R2 Retrospective model	(20)
Duroux et al., 2026	Masked autoencoders for MALDI-TOF spectra; SELFIES/Molformer antimicrobial encodings; ResMLP classifier	Prediction/generalizability	T2 prediction; validation/generalizability; implementation-readiness evidence	Not specified	R3 External/temporal validation	(33)
Li et al., 2024	Multiple ML algorithms; temporal and external validation; AUROC evaluation	Classification/risk stratification	T3 risk stratification; T7 decision support; V3/V4 validation evidence	Feature importance/predictors reported	R3 External/temporal validation	(21)
Lewin-Epstein et al., 2021	L1 logistic regression, gradient-boosted trees, neural network, ensemble; permutation importance and SHAP	Classification/empiric therapy prediction	T2 prediction; T7 clinical decision support	Permutation tests and SHAP	R2 Retrospective model	(22)
Nigo et al., 2024	PyTorch_EHR deep learning time-series model compared with logistic regression and LightGBM	Classification/risk stratification	T3 risk stratification; T7 decision support; external validation	Not reported	R3 External/temporal validation	(23)
Haredasht et al., 2025	LightGBM using demographics, prior resistance, prescriptions, comorbidities, prior microbiology, and local antibiogram features; 5-fold cross-validation and SHAP feature importance.	Classification/decision support	T2 prediction; T7 decision support; stewardship	Feature importance from model	R2 Retrospective model	(24)
Woh et al., 2025	XGBoost and convolutional neural network (CNN) using one-hot encoded AMR gene and antibiotic-resistance features; SHAP interpretation.	Classification/food-safety surveillance	WGS-based AMR prediction for nontyphoidal *Salmonella* surveillance in food-safety and public-health settings.	SHAP values for antibiotic-specific feature contributions	R2 Retrospective model	(26)
Van Camp et al., 2020	Eight XGBoost-based models using coverage of known AMR genes; reliability index; web/standalone predictor	Classification/decision support	T2 prediction; genomic surveillance; clinical decision support	Reliability index	R3 External/temporal validation	(27)
Gao et al., 2024	RF, SVM, XGBoost using k-mer features and feature-selection pipelines	MIC prediction/rapid diagnostic prediction	T2 prediction; genomic surveillance; rapid AST replacement/adjunct	Feature selection/top-ranked 11-mers	R3 External/temporal validation	(28)
Pan et al., 2025	Lasso regression to identify WGS resistance features; mNGS-AST prediction model using key genomic markers and sequencing-depth thresholds.	Rapid prediction/clinical decision support	T2 prediction; T7 decision support; rapid diagnostics	Resistance feature identification	R3 External/temporal validation	(29)
Zhang et al., 2022	Risk scoring integrating human accessibility, mobility, pathogenicity, clinical availability; ML mapping of marine AMR threats	Risk mapping/hotspot prediction	T6 hotspot/risk mapping; T8 One Health/environmental surveillance	Risk-index integration	R2 Retrospective model	(30)
Secker et al., 2026	Shotgun metagenomics; diversity/correlation/geographic analyses	Tracking/geographic mapping	T1 tracking; T5 early warning; T6 hotspot mapping; LMIC/One Health	Not applicable	R2 Retrospective model	(40)
Knight et al., 2024	HT qPCR targeting 73 ARGs compared with metagenomic sequencing	Tracking/method comparison	T1 tracking; T8 One Health/environmental; surveillance methodology	Not applicable	R2 Retrospective model	(41)
López-Cortés et al., 2025	SVM, RF, logistic regression, CatBoost; 10-fold cross-validation; transfer learning; SHAP	Rapid prediction/biomarker identification	T2 prediction; T7 decision support; explainable biomarkers	SHAP values	R2 Retrospective model	(34)
Feucherolles et al., 2022	MALDI-TOF protein spectra plus ML classifiers; on/off-plate extraction comparison; biomarker identification	Rapid screening	T2 prediction; D11 food-chain; T8 One Health	Putative AMR biomarker identification	R2 Retrospective model	(35)
Wiesmann et al., 2025	LR, MLP, SVM, RF, LGBM, and XGB; 10 random 80/20 splits with inner 5-fold cross-validation; external DRIAMS-A comparison; prospective 18-month performance monitoring.	Validation/temporal drift assessment	MALDI-TOF AMR prediction, external transportability assessment, and temporal model-drift/retraining evidence.	Not central	R4 Prospective/operational	(36)
Jeffrey et al., 2020	ARIMA, ETS, neural-network autoregression, Teodoro/EMD-KNN forecasting, naive forecasting, and INLA geospatial modelling.	Forecasting/geospatial modelling	T4 forecasting; T6 geospatial/spatiotemporal modelling	Not reported	R3 External/temporal validation	(42)
Csorba 2025a	Isolation Forest anomaly detection; interpretable driver ranking	Anomaly detection/early warning	T5 early warning; T3 risk stratification; One Health/environmental risk	Feature contribution/interpretable driver ranking	R2 Retrospective model	(43)
Csorba 2025b	XGBoost/SVC exploratory classification; Generalised Naive Bayes; Bayesian Network for conditional dependencies	Early-warning/probabilistic risk modelling	T5 early warning; T8 One Health; D11 food-chain/agriculture	Bayesian network interpretability/feature dependencies	R2 Retrospective model	(44)
Kim et al., 2023	SARIMA compared with ARMA, ARIMA, and other baselines	Time-series forecasting	T4 forecasting; D10 animal/veterinary; One Health	Not reported	R2 Retrospective model	(45)
Martínez-Agüero et al., 2022	Feature selection; interpretable recurrent neural networks; fuzzy linguistic explanations; clinician validation	Clinical time-series risk prediction	T3 risk stratification; T5 early warning; T7 decision support	Fuzzy linguistic explanations; interpretable feature selection	R2 Retrospective model	(50)
Kanjilal et al., 2020	Logistic-regression susceptibility models with constrained decision algorithm	Prediction and decision rule	Antimicrobial stewardship decision support	Yes; interpretable decision rule	R2 Retrospective model	(51)
Shapiro Ben David et al., 2025	ML resistance forecasting integrated in a deployed order set	Forecasting and decision support	Deployed stewardship decision support	Limited model explainability	R4 Prospective/operational	(52)
Nguyen et al., 2019	XGBoost regression on k-mer features	MIC prediction	Genomic AMR surveillance (in silico antibiogram)	Yes; genomic feature selection	R2 Retrospective model	(53)
Yagimoto et al., 2024	ProteinBERT protein language model	ARG mechanism classification	Genomic resistance-mechanism prediction	Yes; attention analysis of binding sites	R3 External/temporal validation	(54)
Munk et al., 2022	Metagenomic analytics and statistical resistome modelling	Resistome mapping and transmission analysis	Global AMR tracking and One Health surveillance	Yes; ARG and flanking-sequence analysis	R2 Retrospective model	(55)
Oonsivilai et al., 2018	Random forest and comparator classifiers	Empiric susceptibility prediction	Locally-tailored empiric-therapy decision support	Yes; variable importance	R2 Retrospective model	(60)

AMR, antimicrobial resistance; AI, artificial intelligence; CNN, convolutional neural network; GBDT, gradient boosting decision tree; KNN, k-nearest neighbours; LDA/QDA, linear/quadratic discriminant analysis; LR, logistic regression; ML, machine learning; MLP, multilayer perceptron; RF, random forest; SHAP, SHapley Additive exPlanations; SVM, support vector machine. Readiness categories: R1, conceptual/contextual; R2, retrospective model; R3, temporally or externally validated; R4, prospectively evaluated or operationally deployed. Readiness codes describe implementation maturity within this evidence set and should not be interpreted as field-wide prevalence. Function codes: T1, tracking; T2, prediction; T3, risk stratification; T4, forecasting; T5, early warning; T6, hotspot or spatial mapping; T7, decision support; T8, One Health integration. Domain codes: D10, animal/veterinary; D11, food-chain/agriculture.

### Surveillance-intelligence functions

Across the 41 studies mapped in Figures 3, 4, prediction and risk stratification were the most frequently represented functions, with additional evidence for tracking, forecasting, early warning, hotspot mapping, decision support, and One Health integration. In Figure 3, EHR and clinical data mapped most strongly to risk stratification and decision support, and WGS and genomic data mapped primarily to prediction. MALDI-TOF and microbiology or LIS data mapped to prediction and decision support, while wastewater, metagenomic, environmental, and geospatial data contributed mainly to tracking, early warning, hotspot mapping, and One Health integration. Because individual studies could contribute to multiple data-source and function categories, the cells in Figure 3 are not mutually exclusive and should not be summed.

**Figure 3:**
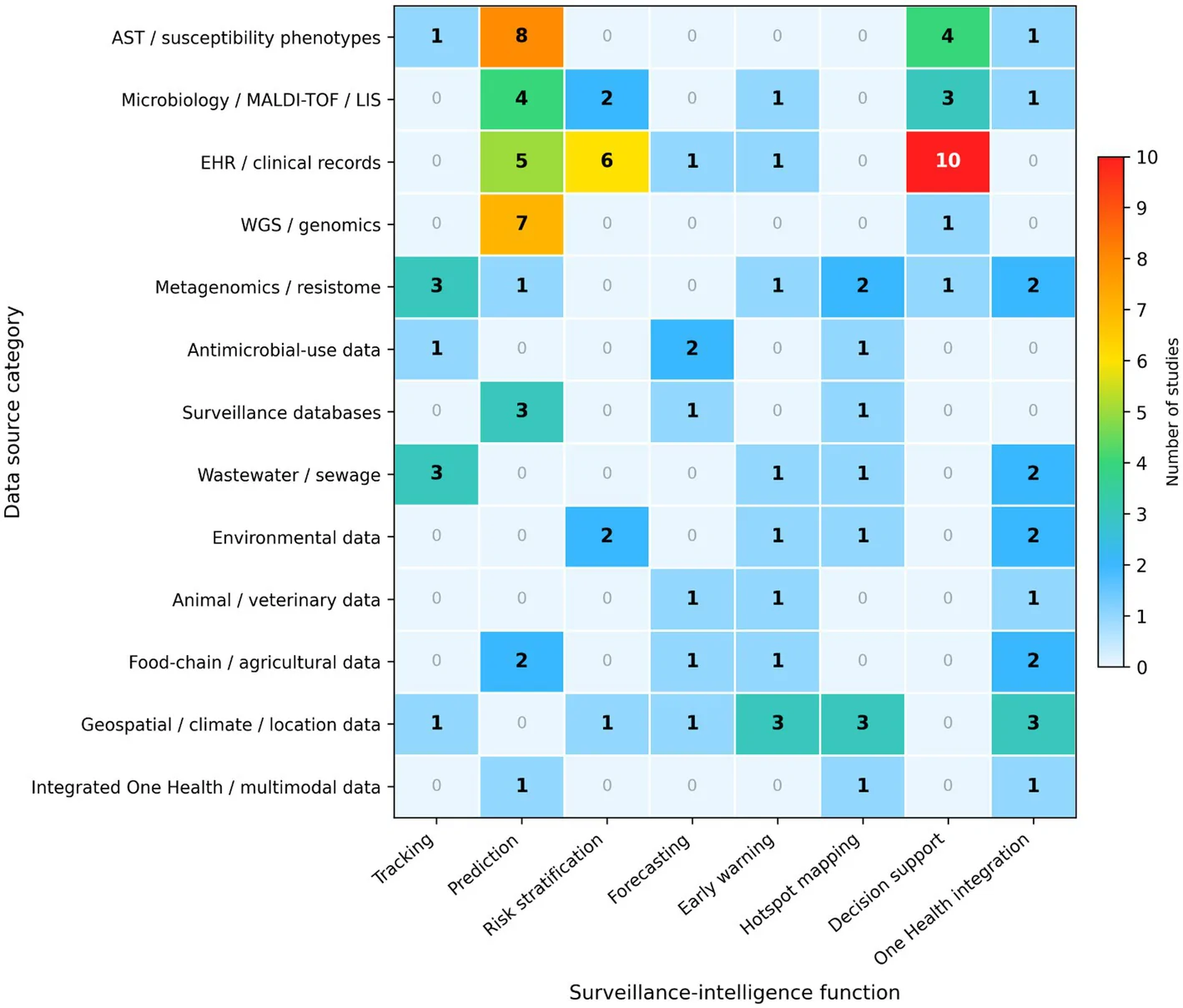
Evidence map of AMR surveillance-intelligence functions by data source. Counts are based on 41 studies: 40 core empirical studies plus one supportive preprint, excluding one contextual review/framework source. Studies contribute to more than one data-source category and more than one surveillance-intelligence function; therefore, cells are not mutually exclusive and should not be summed to estimate the total number of included studies. Rows are data-source categories and columns are surveillance-intelligence functions; Each cell gives the number of studies linking that data source to that function, and the cell colour is proportional to this count (pale = few studies; red = the maximum).

**Figure 4:**
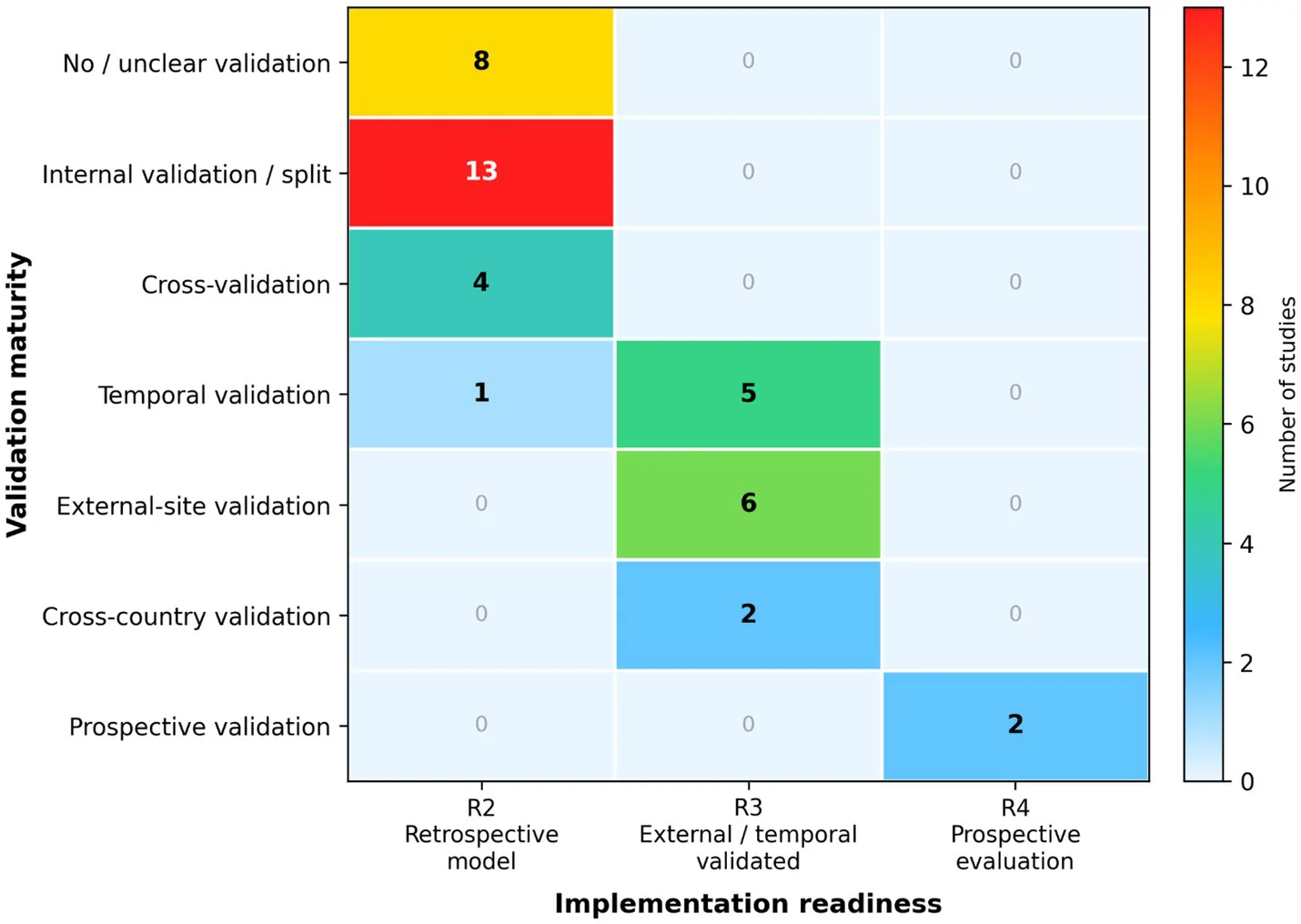
Validation maturity and implementation-readiness map. Cells show synthesis-set counts. Counts are based on 41 studies: 40 core empirical studies plus one supportive preprint, excluding one contextual review/framework source. Counts should not be interpreted as prevalence in the entire AMR machine-learning literature. Rows are validation-maturity levels (from no or unclear validation up to prospective validation) and columns are implementation-readiness stages (R2, retrospective model development; R3, external or temporal validation; R4, prospective evaluation); each cell gives the count of studies at that combination, colour-scaled by count, and the cells sum to 41.

Forecasting and early-warning evidence was present but heterogeneous. Forecasting studies included hospital AMR prevalence prediction, early susceptibility forecasting across bacteriology workflow stages, and time-series/geospatial resistance forecasting (14, 16, 42). Early-warning studies included environmental anomaly detection, broiler-chain early-warning modelling, and interpretable clinical time-series modelling for early multidrug-resistance prediction in ICU patients (43, 44, 50).

### Validation and performance

Validation maturity varied substantially (Table 4). In the validation and implementation-readiness map (Figure 4), eight were descriptive surveillance, forecasting, or methods studies without a discriminative classifier and were not assigned a model-validation tier. Among the remainder, 13 were coded as internal validation or split, four as cross-validation, six as temporal validation, six as external-site validation, two as cross-country validation, and two as prospective validation. These counts are restricted to the synthesis mapping set and should not be interpreted as prevalence in the entire AMR machine-learning literature. Performance metrics were not pooled because the data sources, AMR outcomes, pathogens, validation designs, and class imbalance differed across studies.

**Table 4:** Validation, performance, and limitations.

Studies	Validation/testing	Performance/key result	Validation category	Limitations/interpretation notes	References
Ardila et al., 2025	Review assessed included studies; PROBAST risk-of-bias framework used; k-fold cross-validation was common among included studies	GBDT, RF and XGBoost were top-performing model families; GBDT mean AUROC 0.80 vs. LR mean 0.68; RF mean AUROC 0.75 vs. LR mean 0.71	V0 None/unclear	Review-level evidence; conclusions constrained by retrospective designs, nonstandard data processing, and lack of randomized/real-world validation in included studies.	(10)
Ferrari et al., 2024	80/20 stratified train-test split; no external validation reported in article	MOSR outperformed conventional ML for BSI/AMR detection; reported higher AUROC/F1 stability in imbalanced and balanced scenarios; F1 values 0.42 and 0.44 for chosen MOSR individuals	V1 Internal split	Single-centre, complete-case reduction from >5,000 to 1,142 patients; binary AMR outcome; proof-of-concept; external validation required before deployment.	(12)
Diaz et al., 2025	80/20 train-test split; internal validation; no external geographic validation reported.	Binary RF/XGBoost AUC ROC around 0.71; RF NPV about 93.2%; multi-class RF AUCs: ESBL 0.95, CRE 0.95, AmpC 0.79, MRSA 0.91, VRE 0.95. SVI and ADI ranked among important predictors in RF models.	V1 Internal split	Single metropolitan region; class imbalance; possible duplicate care across systems; socioeconomic indices from 2020 may not match full 2006–2023 period; external validation needed.	(13)
Ureña et al., 2024	Temporal validation on 2020 dataset; comparison across four bacteriology-process stages: sampling, direct examination, positive culture, species identification.	Mean AUROC rose from 0.594 at sampling to 0.708 at direct examination, 0.773 at culture and 0.847 at species identification. NN mean AUROC 0.823; Bayesian model AUROC reached 0.918 at species stage.	V1 Internal split	Single-center proof-of-concept; requires prospective workflow testing and clinical-impact evaluation before routine use.	(14)
Hardan et al., 2024	Internal train/test experiments across different prediction windows and data modalities; no prospective or external validation reported.	MMIM achieved AUROC 0.76 and AUPR 0.1825 for Gentamicin T = 4; AUROC 0.66 and AUPR 0.227 for Gentamicin T = 3; *P. aeruginosa* AUROC 0.69 with AUPR 0.30.	V1 Internal split	Class imbalance; low AUPR; exploratory/developmental; no clinical deployment or external validation.	(15)
Hendriksen et al., 2019	Global cross-sectional surveillance with duplicate samples at selected sites; predictive modelling to estimate AMR gene abundance across countries.	Detected 1,625 AMR genes across 408 gene groups; AMR abundance varied by continent; country-level variables explained up to 89% of observed variation; predicted AMR in 259 countries/territories.	V0 None/unclear	Short-read metagenomics limits host/genetic-context inference; sewage signals combine human/environmental sources; not a clinical decision-support model.	(37)
Weis et al., 2022	80/20 train-test splits stratified by class and patient case; cross-site DRIAMS-A-D validation; temporal validation with sliding training windows; retrospective clinical review of 63 invasive-infection cases.	Selected AUROC values: *S. aureus* oxacillin 0.80, *E. coli* ceftriaxone 0.74, *K. pneumoniae* ceftriaxone 0.74. Treatment recommendation would have changed in 9/63 cases and been beneficial in 8/9 of those changed cases.	V4 External cohort	Predictors were not fully transportable across sites; performance declined with temporal distance; authors recommend local training and regular retraining/re-certification before clinical deployment.	(31)
Ren et al., 2024	70/30 stratified train/test splits; 5-fold CV during tuning; external validation on DRIAMS-B–D where available.	Tuned models achieved mean AUROC 0.80–0.95; best classifiers were commonly LightGBM or SVM; AUPRC up to 0.97.	V3 Temporal/ext-site	Retrospective; dependent on DRIAMS data quality and spectra preprocessing; no prospective clinical-impact study.	(32)
Vihta et al., 2024	Temporal evaluation at NHS-Trust level across financial years 2016/17 to 2021/22; 22 pathogen-antibiotic combinations; compared against a previous-value baseline.	XGBoost best overall and particularly useful when Trust-level AMR prevalence changed substantially; previous value also strong when prevalence stable	V3 Temporal/ext-site	Short time series, annual aggregation, high-income setting, mainly bloodstream infections	(16)
Valavarasu et al., 2025	80:20 train-test split on Pfizer ATLAS surveillance data (917,049 isolates; 589,998 genotype-linked).	XGBoost achieved AUC 0.96 phenotype-only and 0.95 phenotype+genotype; antibiotic tested was the dominant feature	V1 Internal split	Dependent on availability/quality/granularity of ATLAS-like data; retrospective validation	(17)
Nsubuga et al., 2024	Trained on English whole-genome data and externally validated against independent African datasets (Uganda, Nigeria, Tanzania).	Performance varied by drug; England-trained models had mixed generalizability to African datasets; LR performed strongly for ampicillin in African validation	V5 Cross-country	Transferability inconsistent; accuracy lower for some external drug-country combinations	(25)
Feng et al., 2025	Internal model evaluation on cross-sector One Health samples (human, food, environment; *n* = 592, China).	40 ARG types and 743 subtypes identified; food/environment carried higher ARG loads than human fecal samples; ML predicted carbapenem-resistant strain presence	V1 Internal split	Regional single-city study; observational; requires careful interpretation of transmission directionality	(38)
Feng B et al., 2025	National cross-sectional metagenomic dataset (39 sites, 22 provinces); internal model evaluation.	Southeastern China showed the highest ARG pollution; antibiotics explained 24.16% of ARG variation; 24.58% of detected ARGs posed potential health risk; machine learning predicted higher ARG risk in areas of intensive human activity.	V0 None/unclear	Cross-sectional design; no external or temporal validation; relative-abundance metagenomic inference; machine-learning algorithm detail limited in the source.	(39)
Yu et al., 2026	Internal test set (development *n* = 1,666 including 223 CRPA) plus independent external validation cohort (*n* = 471); LASSO selection, SHAP, decision-curve analysis, SMOTE.	RF internal test AUC 0.837 and external AUC 0.898; high specificity but modest sensitivity, supporting rule-in use	V4 External cohort	Designed as adjunct/rule-in, not standalone screen; negative predictions cannot rule out CRPA	(18)
Mutahwa et al., 2025	80/20 train-test split, grid search, and 10-fold stratified cross-validation; test set *n* = 811.	SVM was best: accuracy 72.08%, precision 73.25%, recall 79.78%, F1 0.76, AUC-ROC approximately 0.79.	V1 Internal split	medRxiv preprint; not certified by peer review; single public-sector hospital; routine laboratory reports lacked key clinical detail and acquisition-site information; generalizability beyond Gweru/Zimbabwe uncertain.	(19)
Zhao et al., 2025	Internal train-test split on 825 ICU patients (375 with MDRO); six ML algorithms compared.	RF best with AUC 0.83 and accuracy 76.7%; urinary catheterization, ventilator use, and antibiotic exposure key risk factors	V1 Internal split	Single-center retrospective study; integration into hospital systems proposed rather than operationally evaluated	(20)
Duroux et al., 2026	Generalizability evaluated across unseen time periods and across hospitals using DRIAMS (54,283 spectra; 65 antimicrobials).	MAE + SELFIES improved AUPRC by 4% on unseen time periods; MAE + Molformer improved by 10% across hospitals	V3 Temporal/ext-site	Still benchmark/development study; no prospective clinical deployment	(33)
Li et al., 2024	Temporal split with cross-database testing on PLAGH-ICU (*n* = 3,536) and MIMIC-IV (*n* = 34,923).	Temporal AUROC 0.786 for PLAGH-ICU and 0.744 for MIMIC-IV; external validation showed reduced performance	V5 Cross-country	Performance decline under external validation; needs refinement across clinical contexts	(21)
Lewin-Epstein et al., 2021	Held-out validation on EMR data; per-antibiotic sample sizes 2,235 to 4,360.	Samples by antibiotic ranged 2,235–4,360; ensemble auROC improved when bacterial species identity included	V3 Temporal/ext-site	Single-center retrospective; needs implementation/prospective evaluation	(22)
Nigo et al., 2024	Internal development (Memorial Hermann) with external validation on MIMIC-IV (393,713 culture events); patients stratified into high/medium/low risk.	PyTorch_EHR AUROC 0.911 development and 0.859 external; stratified patients into high/medium/low risk	V3 Temporal/ext-site	Predicts MRSA culture positivity, not all AMR; clinical impact not prospectively tested	(23)
Haredasht et al., 2025	Temporal holdout: models trained on 2009–2021 encounters and tested on 2022–2023 encounters; 5-fold cross-validation used during model development.	AUROC 0.74–0.78 across five antibiotic models; cefazolin highest AUROC 0.78; specificity supported potential antibiotic de-escalation at selected thresholds.	V1 Internal split	Single-center Stanford cohort; positive cultures only; no external validation; training and testing periods span COVID-era practice changes; EHR interoperability limited outside-system data.	(24)
Woh et al., 2025	80/20 training/test split; XGBoost grid search with fivefold cross-validation; CNN validation reported. Full text notes data imbalance and public-database source constraints.	High reported accuracy for both models: XGBoost 0.97625; CNN 0.9904.	V2 Single-set CV	Public database overrepresented non-resistant isolates; few animal/environmental samples; sparse data from several ASEAN countries; potential feature overreliance for some antibiotics.	(26)
Van Camp et al., 2020	Cross-validation on 946 balanced samples (915 for the eight models); in-house validation set (*n* = 19).	Models showed high performance and stability under class imbalance; provided in silico antibiogram for eight drugs	V4 External cohort	Relies on known gene coverage; phenotype–genotype relationships complex	(27)
Gao et al., 2024	Independent test set; essential- and category-agreement assessed against reference phenotype.	Average essential agreement >90.90%, category agreement >95.29%, independent test average accuracy 0.96; predictions within 10 min	V4 External cohort	Species-specific; independent validation but not operational deployment	(28)
Pan et al., 2025	WGS model evaluated across five antibiotics; mNGS-AST model clinically validated using 104 paediatric respiratory specimens.	WGS model AUCs: IPM 91.25%, MEM 89.69%, CIP 88.17%, LEV 91.01%, SXT 90.93%; mNGS-AST accuracy 95.18–100%; turnaround reduced by 69.64 h.	V4 External cohort	Paediatric hospital validation; some specimens lacked sufficient coverage for clear susceptibility calls; quinolone resistance features missed in some cases; broader general-hospital validation warranted.	(29)
Zhang et al., 2022	Descriptive global marine ARG health-risk mapping (random-forest component).	23.78% of ARGs posed health risk; ML mapped global marine antibiotic resistance threats with >75% accuracy	V0 None/unclear	Risk score depends on assumptions/available metagenomic data; environmental-to-human risk inference	(30)
Secker et al., 2026	Metagenomic profiling across urban and rural wastewater sites (descriptive surveillance).	Detected 871 bacterial genera and 606 unique ARGs; resolved urban–rural AMR/pathogen reservoir structure	V0 None/unclear	Retrospective sampling; no direct clinical outcome linkage	(40)
Knight et al., 2024	Comparison of metagenomics and high-throughput qPCR across 47 wastewater treatment plants (descriptive surveillance).	Metagenomics detected 545 genes and provided host/risk context; qPCR quantified all targeted genes with higher sensitivity	V0 None/unclear	Method comparison; not a predictive ML paper; data availability on request	(41)
López-Cortés et al., 2025	Single-centre internal validation on 2,229 VITEK-MS spectra.	CatBoost AUROC 0.91 and F1 0.78 for *E. coli* ciprofloxacin; spectral peaks identified via SHAP	V1 Internal split	Single-center/platform; transfer learning suboptimal; needs multi-site validation	(34)
Feucherolles et al., 2022	Internal validation on a One-Health research collection of 340 isolates (9,180 MALDI-TOF spectra reduced to 1,020 reference profiles).	High performance for susceptible and ciprofloxacin/tetracycline-resistant isolates; maximum sensitivity 92.3% and precision 81.2%	V1 Internal split	Performance varied by resistance; screening tool rather than definitive AST	(35)
Wiesmann et al., 2025	10 random train-test splits with inner 5-fold cross-validation; cross-site validation between Germany and DRIAMS-A Switzerland; prospective 18-month performance observation.	Best local AUROCs: oxacillin/*S. aureus* RF 0.85, ciprofloxacin/*E. coli* XGB 0.83, piperacillin-tazobactam/*K. pneumoniae* XGB 0.81; AUROC declined over 18 months.	V6 Prospective	Performance declined when training/test data differed by location/time; models require local training, standardised spectra/AST data, and regular retraining before routine deployment.	(36)
Jeffrey et al., 2020	Temporal holdout forecasting using EARS-Net European annual data and England quarterly CCG data; RMSE and prediction-interval accuracy used for evaluation.	Naive forecasting performed best for coarse annual European data; ETS performed best for finer quarterly English data; geospatial INLA did not improve average accuracy but helped examine covariates.	V3 Temporal/ext-site	Annual national surveillance data too coarse for accurate forecasting; finer spatiotemporal data needed.	(42)
Csorba 2025a	Internal anomaly-detection evaluation on high-level environmental indicators.	Pesticide use, population density, land-use change, and fertilizer application dominated anomaly scores	V0 None/unclear	Anomaly-based proxy model; does not directly validate against AMR outcomes in all cases	(43)
Csorba 2025b	Single-set cross-validation of a graph-based and Bayesian broiler-chain early-warning model.	Pesticides, land use, and precipitation emerged as key features; resistance probability was highly context-dependent	V2 Single-set CV	Hypothesis-generating high-level indicator approach; requires ground-truth and prospective validation	(44)
Kim et al., 2023	Time-series forecasting evaluated against five baselines (descriptive).	SARIMA outperformed five baselines for future AMR burden prediction	V0 None/unclear	Food-animal production focus; may not generalise to clinical settings without recalibration	(45)
Martínez-Agüero et al., 2022	Single-set cross-validation of interpretable ICU time-series models with intelligent feature selection.	Models balanced performance and interpretability for early prediction of antimicrobial multidrug resistance; clinicians valued explainability	V2 Single-set CV	Limited/imbalanced data and irregular time-series; performance details require table extraction if used quantitatively	(50)
Kanjilal et al., 2020	Retrospective temporal test cohort; 3,629 patients (2014–2016)	67% reduction in second-line use and 18% fewer inappropriate therapies versus clinicians	V1 Internal split	Single US health system; retrospective counterfactual; generalizability untested	(51)
Shapiro Ben David et al., 2025	Operational deployment; 19,287 culture-paired cases	Antibiotic mismatch 8.9% versus 14.2% when followed; 80.5% reduction in ciprofloxacin use	V6 Prospective	Single organisation; observational; possible confounding by adoption	(52)
Nguyen et al., 2019	Single-set cross-validation; temporal stability check	95% accuracy within plus or minus one doubling dilution across 15 antibiotics	V2 Single-set CV	One surveillance dataset; no external clinical or prospective validation	(53)
Yagimoto et al., 2024	Single-set cross-validation including low-homology test	Outperformed state-of-the-art, including low-similarity ARGs	V4 External cohort	Benchmark datasets; no clinical or prospective evaluation	(54)
Munk et al., 2022	Single-set analytic; regional pattern analysis	Mapped regional resistomes and ARG genetic contexts across 101 countries	V0 None/unclear	Descriptive surveillance; not a clinical prediction model	(55)
Oonsivilai et al., 2018	Internal train/test split	AUC 0.80 (ceftriaxone) and 0.74 (ampicillin-gentamicin)	V1 Internal split	Single LMIC centre; small sample; internal validation only	(60)

AUROC, area under the receiver operating characteristic curve; AUPRC, area under the precision-recall curve; AMR, antimicrobial resistance; ML, machine learning. Validation categories: V0 = none or unclear; V1 = internal train-test split; V2 = single-set cross-validation; V3 = temporal or external-site validation; V4 = external-cohort validation; V5 = cross-country validation; V6 = prospective or operational validation. Performance values are not pooled because studies differ by pathogen, outcome, dataset, model, validation design, and clinical or environmental context.

Several studies reported strong discrimination or classification performance, but the performance metrics were not directly comparable because the data sources, outcomes, pathogens, validation designs, and class imbalance varied. Diaz et al. reported RF/XGBoost and multiclass random-forest performance for blood-culture AMR outcomes (13). Ureña et al. reported increasing area under the receiver operating characteristic curve (AUROC) across bacteriology workflow stages (14). Yu et al. reported internal and external validation for CRPA risk stratification (18). Pan et al. reported WGS- and metagenomic next-generation sequencing (mNGS)-based rapid prediction for *Enterobacter cloacae* complex with area under the curve (AUC) values of 88.17–91.25% across five antibiotics and >95% accuracy in 104 clinical specimens (29).

### Implementation readiness and limitations

Most studies remained at retrospective model-development or retrospective validation stages. In the implementation-readiness map, 26 studies were classified as retrospective model development, 13 as retrospective models with external or temporal validation, and two as prospective evaluation or operational deployment. The evidence therefore supports growing technical capability for AMR surveillance intelligence, but it does not yet show broad operational deployment into real-time surveillance systems, public-health dashboards, antimicrobial stewardship workflows, or One Health response platforms. Common limitations included single-centre design, retrospective analysis, internal validation only, limited external transportability, class imbalance, one retained supportive preprint record, lack of prospective clinical-impact evaluation, and limited operational deployment evidence.

Feng B et al. provide environmental AMR surveillance evidence in a national-scale study of rural domestic sewage across 39 sites in 22 Chinese provinces. The study profiled 702 antibiotic resistance gene (ARG) subtypes by metagenomic sequencing and applied machine learning to predict ARG health risk (39).

### Synthesis of evidence gaps

The evidence base demonstrates broad technical activity across clinical, laboratory, genomic, environmental, wastewater, animal, food-chain, and One Health data streams (Table 5). The clearest evidence clusters were EHR-based risk prediction, genomic or WGS-based AMR prediction, MALDI-TOF rapid resistance prediction, and wastewater/metagenomic surveillance. The least mature areas were prospective deployment, real-time early-warning systems, operational dashboards, implementation-effect studies, model maintenance under temporal drift, and cross-sector One Health response platforms. Overall, the evidence supports movement from passive AMR monitoring toward surveillance intelligence, but most studies remain closer to retrospective analytics than routine operational response systems.

**Table 5:** One Health, sector, geography, and equity relevance.

Studies	Sector/domain	Country/region	One Health category	LMIC/equity	Surveillance relevance	Limitations/interpretation notes	References
Ardila et al., 2025	Human	Global	0 None	Partial	Contextual evidence for AMR prediction in real-world healthcare settings	Review-level evidence; conclusions constrained by retrospective designs, nonstandard data processing, and lack of randomized/real-world validation in included studies.	(10)
Diaz et al., 2025	Human	United States	0 None	Equity-relevant (socioeconomic deprivation indices); high-income setting	Patient-level risk stratification and empirical antibiotic decision support while awaiting culture/susceptibility results.	Single metropolitan region; class imbalance; possible duplicate care across systems; socioeconomic indices from 2020 may not match full 2006–2023 period; external validation needed.	(13)
Hardan et al., 2024	Human health	USA/MIMIC-IV	0 None	No	Real-time patient-level AMR prediction from routinely available EHR data; potential EHR workflow integration.	Class imbalance; low AUPR; exploratory/developmental; no clinical deployment or external validation.	(15)
Hendriksen et al., 2019	Environmental/global	60 countries	3 Integrated One Health analytics	High global relevance	Global AMR tracking, environmental surveillance, geographic prediction and population-level early-warning substrate.	Short-read metagenomics limits host/genetic-context inference; sewage signals combine human/environmental sources; not a clinical decision-support model.	(37)
Weis et al., 2022	Human health/rapid diagnostics	Switzerland	0 None	No; high-income routine diagnostic setting	Rapid diagnostic intelligence for earlier resistance inference and antimicrobial stewardship support.	Requires local validation and regular retraining; retrospective clinical case review only, not prospective deployment.	(31)
Ren et al., 2024	Human health	Switzerland	0 None	No	Rapid MALDI-TOF-based resistance profiling and nosocomial infection decision support.	Retrospective; dependent on DRIAMS data quality and spectra preprocessing; no prospective clinical-impact study.	(32)
Nsubuga et al., 2024	Human	England; Uganda; Nigeria; Tanzania	0 None	Yes	T2 prediction; V5 cross-country validation; LMIC/equity relevance	Transferability inconsistent; accuracy lower for some external drug-country combinations	(25)
Feng et al., 2025	One Health	China	3 Integrated One Health analytics	Partial	T8 One Health integration; T6 hotspot/transmission mapping; T2 prediction	Regional single-city study; observational; requires careful interpretation of transmission directionality	(38)
Feng B et al., 2025	Environment	China (39 sites across 22 provinces)	1 Single non-human sector	Partial	National-scale environmental ARG health-risk mapping and hotspot prioritisation for One Health surveillance.	Cross-sectional design; no external or temporal validation; site representativeness constraints remain.	(39)
Mutahwa et al., 2025	Human	Zimbabwe	0 None	Yes	T2 prediction; T1 tracking; LMIC relevance	Preprint; not certified by peer review; single hospital and routine laboratory data limit generalizability.	(19)
Zhao et al., 2025	Human	China	0 None	Partial	T3 risk stratification; T7 decision support; explainable AI	Single-center retrospective study; integration into hospital systems proposed rather than operationally evaluated	(20)
Duroux et al., 2026	Human	Switzerland/DRIAMS diagnostic laboratory network	0 None	Partial	T2 prediction; validation/generalizability; implementation-readiness evidence	Still benchmark/development study; no prospective clinical deployment	(33)
Li et al., 2024	Human health	China; USA MIMIC-IV	0 None	Partial; includes MIMIC-IV external dataset but high-resource hospital focus	T3 risk stratification; T7 decision support; V3/V4 validation evidence	Performance decline under external validation; needs refinement across clinical contexts	(21)
Lewin-Epstein et al., 2021	Human health	Israel	0 None	No	T2 prediction; T7 clinical decision support	Single-center retrospective; needs implementation/prospective evaluation	(22)
Nigo et al., 2024	Human health	USA	0 None	No	T3 risk stratification; T7 decision support; external validation	Predicts MRSA culture positivity, not all AMR; clinical impact not prospectively tested	(23)
Haredasht et al., 2025	Human health	USA	0 None	No	Patient-level empirical antibiotic decision support and stewardship-oriented de-escalation using EHR-derived personalized antibiograms.	Single-center temporal validation; external validation and implementation-impact evaluation needed.	(24)
Woh et al., 2025	Food-chain/public health/genomic surveillance	Hong Kong and ASEAN countries	2 Cross-sector/multisector	Asia/foodborne public-health relevance; includes food, human, environmental, and animal sources but with imbalanced source representation.	WGS-based AMR prediction for *Salmonella* food-safety and public-health surveillance.	Retrospective public-database study; imbalanced resistance labels and sparse data from some countries/source categories.	(26)
Van Camp et al., 2020	Human health	Public NCBI BioSample data	0 None	No	T2 prediction; genomic surveillance; clinical decision support	Relies on known gene coverage; phenotype–genotype relationships complex	(27)
Gao et al., 2024	Human health	China	0 None	No	T2 prediction; genomic surveillance; rapid AST replacement/adjunct	Species-specific; independent validation but not operational deployment	(28)
Pan et al., 2025	Human health	China	0 None	No	T2 prediction; T7 decision support; rapid diagnostics	Paediatric respiratory specimen validation; incomplete reportability for some samples and broader validation needed.	(29)
Zhang et al., 2022	Environmental/global One Health	Global habitats	3 Integrated One Health analytics	Global relevance	T6 hotspot/risk mapping; T8 One Health/environmental surveillance	Risk score depends on assumptions/available metagenomic data; environmental-to-human risk inference	(30)
Secker et al., 2026	Environmental/wastewater	India	2 Cross-sector/multisector	Strong LMIC relevance	T1 tracking; T5 early warning; T6 hotspot mapping; LMIC/One Health	Retrospective sampling; no direct clinical outcome linkage	(40)
Knight et al., 2024	Environmental/wastewater	Wales, UK	2 Cross-sector/multisector	No	T1 tracking; T8 One Health/environmental; surveillance methodology	Method comparison; not a predictive ML paper; data availability on request	(41)
López-Cortés et al., 2025	Human health	Chile	0 None	No	T2 prediction; T7 decision support; explainable biomarkers	Single-center/platform; transfer learning suboptimal; needs multi-site validation	(34)
Feucherolles et al., 2022	Food-chain/One Health	Luxembourg/One Health panel	2 Cross-sector/multisector	No	T2 prediction; D11 food-chain; T8 One Health	Performance varied by resistance; screening tool rather than definitive AST	(35)
Wiesmann et al., 2025	Human health	Germany; Switzerland DRIAMS-A	0 None	No	Rapid MALDI-TOF AMR prediction plus temporal drift/retraining evidence for routine diagnostic surveillance.	Requires local training/retraining; poor transportability across unrelated location/time settings.	(36)
Csorba 2025a	Environment	Global/environmental indicators	1 Single non-human sector	Partial	T5 early warning; T3 risk stratification; One Health/environmental risk	Anomaly-based proxy model; does not directly validate against AMR outcomes in all cases	(43)
Csorba 2025b	Food-chain/animal health	International databases/poultry production	3 Integrated One Health analytics	Partial	T5 early warning; T8 One Health; D11 food-chain/agriculture	Hypothesis-generating high-level indicator approach; requires ground-truth and prospective validation	(44)
Kim et al., 2023	Animal health/food animal production	United States	1 Single non-human sector	Partial	T4 forecasting; D10 animal/veterinary; One Health	Food-animal production focus; may not generalise to clinical settings without recalibration	(45)
Nguyen et al., 2019	Food/human	USA	2 Cross-sector/multisector	No	Genomic food-safety AMR surveillance	Single surveillance dataset	(53)
Munk et al., 2022	Environmental/One Health	101 countries	3 Integrated One Health analytics	Mixed (global)	Global sewage resistome surveillance	Ecological, population-level intelligence	(55)
Oonsivilai et al., 2018	Human	Cambodia	0 None	Yes	LMIC empiric-therapy decision support	Small single-centre dataset	(60)

AMR, antimicrobial resistance; LMIC, low- and middle-income country. One Health category: 0 = none (human or clinical only); 1 = single non-human sector (environmental, animal, or food-chain); 2 = cross-sector or multisector relevance; 3 = integrated One Health analytics across sectors. These categories summarise whether studies incorporated human, animal, food-chain, environmental, wastewater, or cross-sector data streams. Equity relevance reflects extracted information on LMIC settings, socioeconomic variables, or access-related context.

### Reporting completeness and the feasibility-to-deployment gap

Appraisal of the 41 studies against TRIPOD+AI and PROBAST showed a consistent gradient (Figure 5 and Table 6). The synthesis set combines prediction-model studies with descriptive surveillance, forecasting, and methods studies, so markers specific to prediction models were scored as absent for studies without a discriminative classifier. The percentages are therefore reported across all 41 studies as reporting completeness within this heterogeneous set, not as risk-of-bias estimates restricted to prediction models. Markers that are intrinsic to prediction models, such as calibration and discrimination, are necessarily absent for non-prediction studies, and the percentages would be higher if restricted to the prediction-model subset.

**Figure 5:**
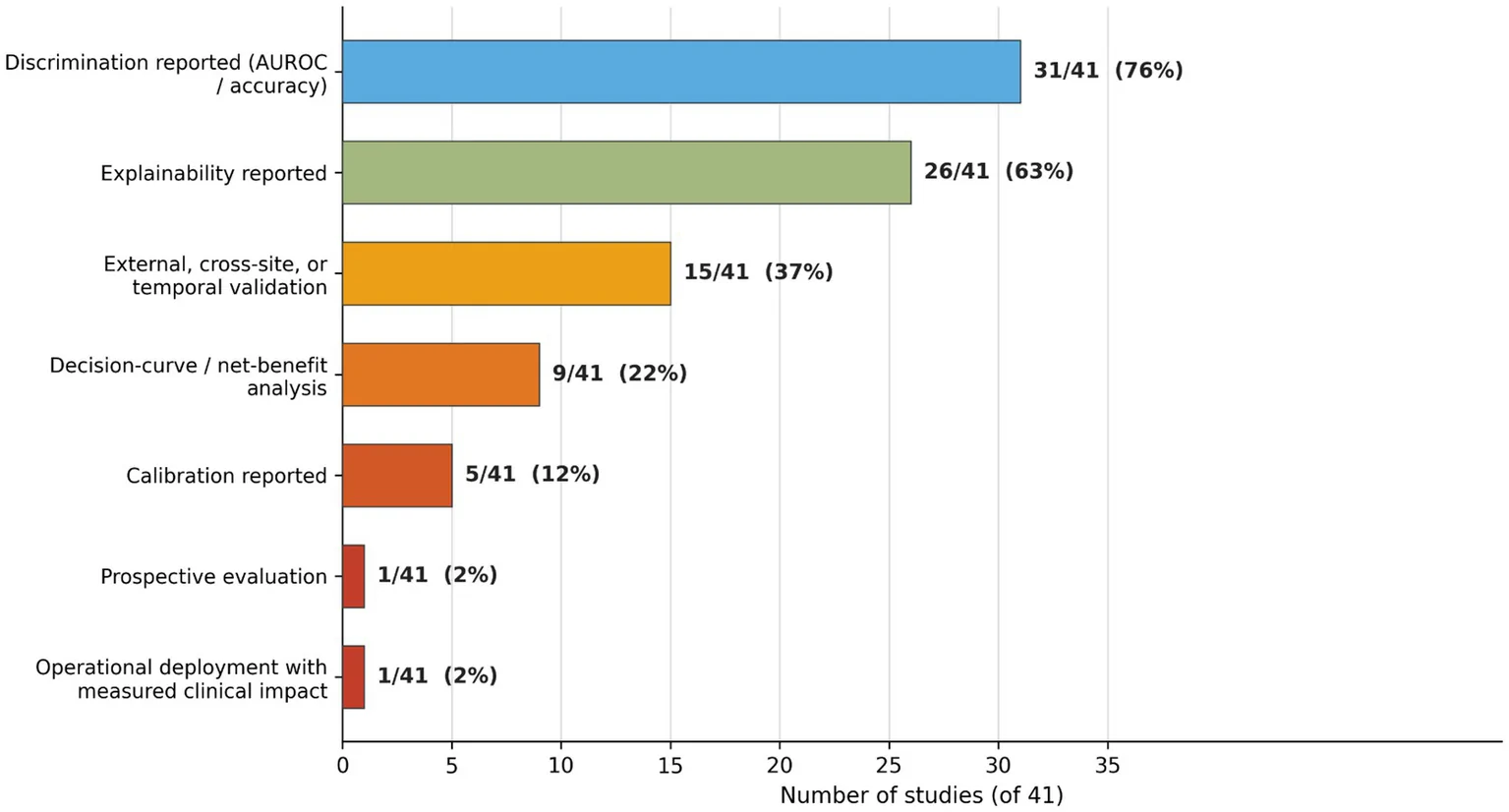
The feasibility-to-deployment gap in machine learning-enabled AMR surveillance. Proportion of the 41 studies reporting each marker, scored from reported results and mapped to TRIPOD+AI and PROBAST. Studies are non-mutually-exclusive. Each horizontal bar gives the proportion (and number, *n*/41) of studies reporting that marker, ordered from the most to the least frequently reported. The descending gradient, from discrimination and explainability to calibration, prospective evaluation, and operational deployment, depicts the gap between technical feasibility and operational readiness.

**Table 6:** Reporting completeness and implementation readiness.

Marker (*n* = 41 studies)	Studies, *n* (%)
Discrimination reported (AUROC or accuracy)	31 (76%)
Explainability reported	26 (63%)
External, cross-site, or temporal validation	15 (37%)
Calibration reported	5 (12%)
Decision-curve or net-benefit analysis	9 (22%)
Prospective evaluation	1 (2%)
Operational deployment with measured clinical or public-health impact	1 (2%)
Implementation stage: retrospective model development	26 (63%)
Implementation stage: external or temporal validation	13 (32%)
Implementation stage: prospective evaluation	2 (5%)

Appraisal of the 41 studies against TRIPOD+AI reporting items and PROBAST risk-of-bias and applicability concepts, scored from reported information.

Discrimination was reported in 31 studies (76%) and explainability in 26 (63%). The markers of deployability were far less common: external, cross-site, or temporal validation in 15 studies (37%), calibration in 5 (12%), decision-curve or net-benefit analysis in 9 (22%), and prospective evaluation in 1 (2%). One study, a deployed outpatient urinary tract infection decision-support tool, demonstrated operational deployment of a live tool with a measured impact on prescribing. One further study reported a measured prescribing effect from a retrospective decision algorithm rather than from live deployment. No other study demonstrated operational deployment with a measured clinical or public-health outcome. By implementation stage, 26 studies (63%) remained at retrospective model development, 13 (32%) reached external or temporal validation, and two (5%) reached prospective evaluation or operational deployment.

## Discussion

### Principal findings

This scoping review shows that machine learning is being applied to AMR surveillance across several distinct data streams, including electronic health records, laboratory information systems, antimicrobial susceptibility testing databases, whole-genome sequencing, metagenomics, wastewater surveillance, MALDI-TOF mass spectrometry, food-chain data, animal-sector data, and environmental indicators. The evidence does not support a single dominant model of AMR surveillance intelligence. Instead, it shows a technically diverse field in which prediction, risk stratification, forecasting, rapid resistance inference, environmental tracking, and One Health mapping are developing in parallel.

The strongest empirical clusters in the evidence set were clinical and EHR-based AMR prediction, genomic or WGS-based AMR prediction, MALDI-TOF-based rapid resistance prediction, wastewater or metagenomic surveillance, and One Health or food-chain early-warning applications. However, the field appears more mature for retrospective prediction than for operational surveillance intelligence. This distinction is central to the manuscript: a technically accurate model is not equivalent to a surveillance system unless it is validated, interpretable, updated over time, and connected to an action pathway.

A central implication is that AMR surveillance intelligence should not be evaluated by model discrimination alone. Several studies reported useful prediction, classification, or forecasting performance, but fewer demonstrated how model outputs changed prescribing, diagnostic triage, infection-prevention action, environmental monitoring, outbreak response, or One Health coordination. The next stage of the field should therefore shift from model development toward implementation science, data governance, calibration monitoring, decision-threshold evaluation, user-facing outputs, and measurable clinical or public-health impact. Within the synthesis set, two studies illustrate that this transition is achievable at different stages of readiness. The first was a retrospective electronic health record-based algorithm for uncomplicated urinary tract infection that would have reduced second-line antibiotic use by 67% and inappropriate therapy by 18% relative to clinicians (51). The second was a deployed urinary tract infection decision-support tool that lowered antibiotic mismatch rates in routine outpatient care (52), the single study demonstrating operational deployment with measured impact.

### Clinical and EHR-based prediction

Clinical and EHR-based studies formed one of the clearest evidence clusters. These studies used intensive-care records, blood-culture data, laboratory information systems, routine clinical variables, complete blood count parameters, longitudinal EHR data, or multimodal EHR inputs to predict AMR, MDRO infection, bloodstream resistance, MRSA culture positivity, or patient-level resistance risk. Examples include EHR/socioeconomic prediction, early susceptibility forecasting, and MRSA time-series prediction (13, 14, 23). This cluster is directly relevant to antimicrobial stewardship because the outputs are potentially actionable at the point of care, including empiric antibiotic selection, early risk stratification, and prioritisation of patients for infection-control action.

### Genomic and WGS-based prediction

Genomic and WGS-based studies show how AMR surveillance can move from phenotypic reporting toward molecularly informed prediction and population-level tracking. Examples include cross-country *E. coli* WGS validation, Gram-negative WGS prediction, *Acinetobacter baumannii* WGS prediction, *Enterobacter cloacae* complex WGS/mNGS prediction, and nontyphoidal *Salmonella* WGS-based food-chain/public-health modelling (25–29). Nsubuga et al. directly addressed generalizability by training AMR prediction models on England *E. coli* WGS data and validating against independent African datasets from Uganda, Nigeria, and Tanzania (25). Woh et al. extended genomic AMR prediction to nontyphoidal *Salmonella* from Hong Kong and Association of Southeast Asian Nations (ASEAN) countries, linking WGS-based XGBoost and convolutional neural network (CNN) prediction to food safety and public-health surveillance (26). The WGS evidence supports genomic surveillance as a major component of AMR surveillance intelligence, but it also shows that genomic prediction depends on data representativeness, lineage structure, phenotype quality, geographic transferability, sequencing infrastructure, and model updating. Whole-genome-based prediction of minimum inhibitory concentrations was demonstrated early and at scale for foodborne *Salmonella* (53), and emerging protein-language-model approaches now predict resistance genes and mechanisms directly from sequence, including for genes with low similarity to known references (54).

### MALDI-TOF and rapid diagnostic analytics

MALDI-TOF plus machine learning was a recurrent rapid-diagnostic theme in the evidence set. Weis et al. trained calibrated classifiers on the DRIAMS database, which combined more than 300,000 MALDI-TOF spectra with more than 750,000 AMR phenotypes. They reported AUROC values of 0.80 for *S. aureus*, 0.74 for *E. coli*, and 0.74 for *K. pneumoniae* in selected clinically important prediction tasks (31). The retrospective 63-case clinical review in Weis et al. suggested that classifier-informed recommendations would have changed treatment in nine cases and been beneficial in eight of them. The authors emphasised, however, that prospective multicenter clinical evaluation would be required before implementation (31). Wiesmann et al. further showed that MALDI-TOF-based AMR classifiers can perform well on local data but lose performance across unrelated location/time settings and over 18 months without retraining (36). These studies are important because MALDI-TOF spectra are already generated in many diagnostic workflows, creating the possibility of extracting AMR-relevant signals before conventional susceptibility results are complete. Surveillance use will still require laboratory-specific validation, platform calibration, drift monitoring, data-quality control, and prospective evaluation.

### Forecasting and early warning

Forecasting and early-warning studies were present but heterogeneous in setting, data granularity, and validation maturity. Vihta et al. used hospital AMR prevalence forecasting at National Health Service (NHS) Trust level, Ureña et al. linked prediction to laboratory workflow timing, and Kim et al. used farm-level AMR data for time-series forecasting (14, 16, 45). Csorba et al. developed environmental anomaly detection and broiler-chain early-warning approaches (43, 44). These studies show proof-of-concept AMR early-warning or forecasting approaches at multiple levels: patient, hospital, farm, environmental, and food-chain systems. However, AMR forecasting should be discussed as an emerging surveillance-intelligence function rather than as a mature operational field.

### Environmental, wastewater, and One Health intelligence

Environmental, wastewater, food-chain, and One Health studies broadened the surveillance-intelligence concept beyond clinical prediction. Hendriksen et al. used untreated sewage metagenomics from 79 sites in 60 countries. Feng et al. analysed cross-sector samples in China. Knight et al. compared wastewater surveillance methods in Wales. Woh et al. linked nontyphoidal *Salmonella* WGS-based AMR prediction to food-chain and public-health surveillance across Hong Kong and ASEAN countries (26, 37, 38, 41). A subsequent global analysis of sewage from 101 countries further mapped the worldwide resistome and its regional patterns (55). These studies are relevant to AMR surveillance because AMR emergence and dissemination involve settings beyond hospitals. However, environmental, food-chain, wastewater, and One Health studies often produce ecological or population-level intelligence rather than direct clinical prescribing recommendations.

Nevertheless, validation remained heterogeneous, and study-by-study interpretation is safer than cross-study performance comparison. Part of this difficulty reflects inconsistent reporting. Adoption of shared standards would make the evidence easier to appraise and compare. Prediction-model development and validation should follow TRIPOD+AI (46), and early-stage clinical evaluation of decision-support systems should follow DECIDE-AI (56).

However, interpretability alone does not prove decision usefulness. A newer class of methods is now entering this space. Foundation models and large language models are being evaluated for antibiotic decision support, but current evidence shows uneven performance. In a comparison of 14 large language models across 60 clinical cases with antibiograms, the best-performing model gave appropriate antibiotic recommendations in about 72 percent of cases, and accuracy fell as case complexity increased (57). Reviews of artificial intelligence for antibiotic prescribing reach a similar conclusion, noting potential value alongside unresolved problems of transparency, explainability, and accountability that currently preclude autonomous use (58). These tools reproduce the central tension of this review: analytic capability has advanced faster than validated, accountable integration into surveillance and stewardship. Studies that apply large language models in this setting should be reported against the dedicated TRIPOD-LLM guideline (59).

These studies show that ML-enabled AMR surveillance intelligence can be relevant beyond high-income hospital settings; for example, locally-tailored machine-learning models have guided empiric antibiotic prescribing in a children’s hospital in Cambodia (60). However, global applicability cannot be assumed from models developed in data-rich environments. Equity also operates at the level of the model. Approaches that draw on socioeconomic or demographic features, such as deprivation-informed prediction (13), can improve discrimination but may encode and amplify existing inequities if they are deployed without fairness assessment. Transparent reporting of subgroup performance and bias, as required by current prediction-model standards (46), is therefore part of equitable AMR surveillance rather than separate from it.

Across all domains, authors should distinguish technical prediction tasks from surveillance-intelligence functions. Read against TRIPOD+AI and PROBAST, the evidence base reports discrimination and explainability far more often than calibration, external validation, or prospective evaluation, which are the domains where prediction-model studies accrue risk of bias and lose applicability. The implication is not that the models are wrong, but that the literature cannot yet tell a surveillance programme which approaches are ready for use. Consistent reporting against TRIPOD+AI (46), early clinical evaluation following DECIDE-AI (56), and explicit linkage of model outputs to measured action would make model quality, calibration, and clinical usefulness directly comparable across studies and close the distance between feasibility and deployment.

### Strengths and limitations

This review has several strengths. First, its scope spans the full One Health AMR data ecosystem, integrating clinical and electronic-health-record models, laboratory-workflow prediction, whole-genome and metagenomic prediction, MALDI-TOF diagnostic analytics, wastewater and environmental surveillance, and food-chain and animal-sector studies within a single synthesis rather than treating any one stream in isolation. Second, it applies an explicit surveillance-intelligence lens that separates technical prediction from validated, deployable intelligence, reframing a literature that is usually read only as a prediction problem. Third, every appraised study was assessed against TRIPOD+AI and PROBAST and mapped onto transparent validation- and implementation-readiness tiers, giving a reproducible, criterion-based account of maturity rather than a narrative impression. Fourth, the synthesis is translated into a usable conceptual framework that links data streams, governance, analytics, outputs, decision support, and feedback. The review also has limitations. First, counts for study characteristics and evidence maps are counts within the synthesis set, not prevalence estimates for the entire AMR machine-learning literature. Second, one retained record remains a supportive preprint rather than peer-reviewed core evidence; it was therefore interpreted cautiously and separated from peer-reviewed full-text evidence. Excluding the single supportive preprint from the appraisal left this gradient essentially unchanged. Third, the heterogeneity of data sources, AMR outcomes, pathogens, validation designs, and performance metrics prevented meta-analysis or algorithm ranking. Fourth, because operational deployment evidence was limited, the review identifies implementation gaps but cannot estimate the effect of ML-enabled AMR surveillance on prescribing, infection control, environmental intervention, or public-health policy. Fifth, eligibility was restricted to English-language publications indexed in three databases (PubMed/MEDLINE, Scopus, and Web of Science) and supplemented by citation searching. Relevant studies published in other languages may therefore have been missed, and a language bias cannot be excluded.

### Proposed One Health AMR surveillance intelligence framework

Based on the synthesis set, the reviewed studies mapped onto six functional layers of AMR surveillance intelligence (Figure 6) namely, One Health data streams, harmonization and governance, machine-learning analytics, intelligence outputs, decision support and action, and a learning feedback loop. This framework separates data generation from actionable surveillance intelligence. In this structure, AMR data become surveillance intelligence only when they are harmonised, analysed with validated and interpretable methods, translated into decision-relevant outputs, and linked to action pathways with feedback for model updating and surveillance improvement.

**Figure 6:**
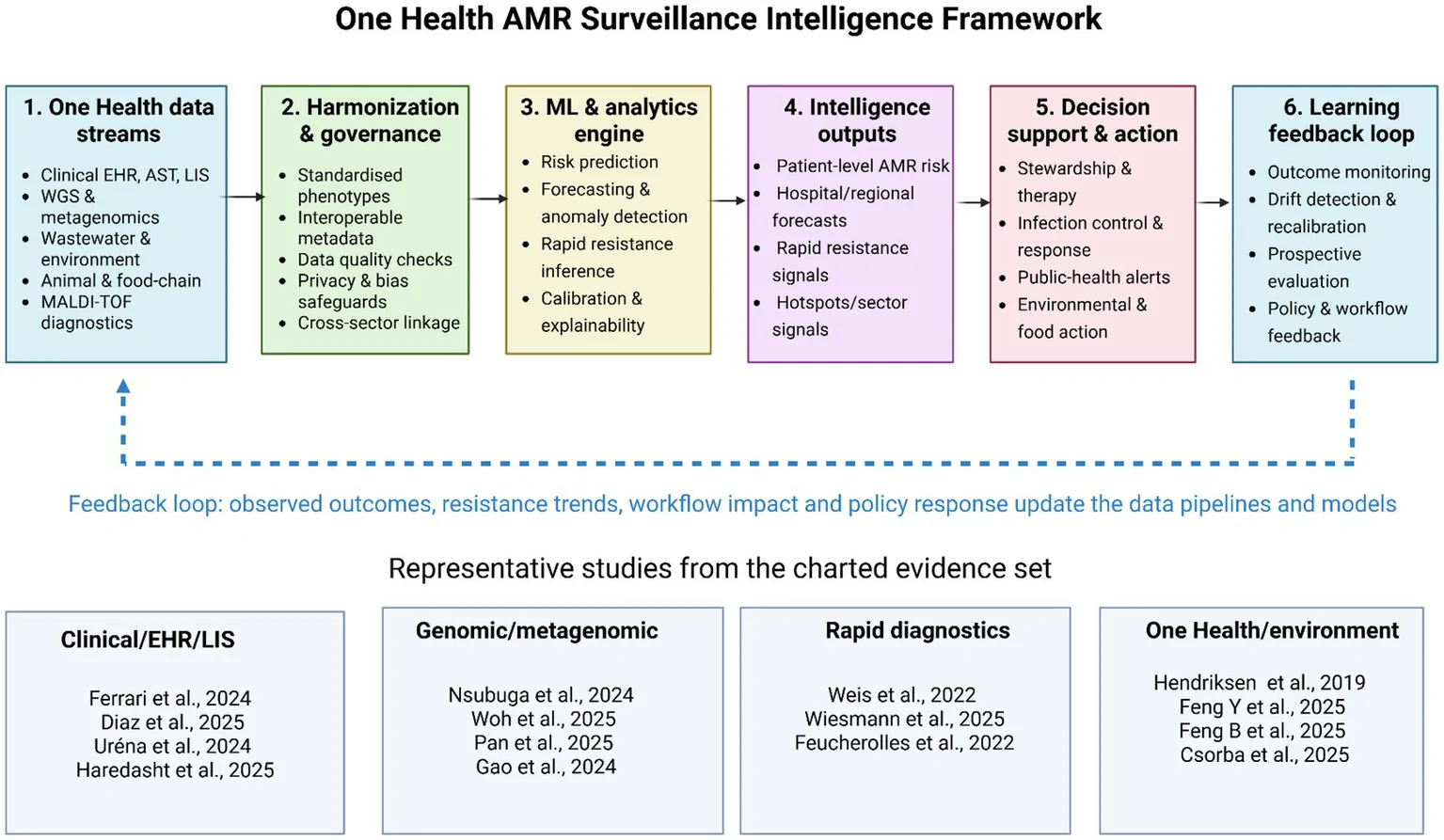
One Health AMR Surveillance Intelligence Framework. The framework is read from left to right across six layers: (1) One Health data streams; (2) harmonization and governance; (3) the machine-learning and analytics engine; (4) intelligence outputs; (5) decision support and action; and (6) a learning feedback loop, with the dashed arrow returning feedback (outcomes, resistance trends, workflow impact, and policy response) to update the data pipelines and models. Representative evidence anchors (Author et al., Year) are grouped by domain beneath the schematic; the figure is a conceptual synthesis and the anchors are supporting examples, not evidence that all framework stages were operationally deployed.

The first layer consists of One Health data streams. Clinical and hospital studies used electronic health records, laboratory information systems, antimicrobial susceptibility testing data, intensive-care records, blood-culture data, complete blood count variables, and microbiology workflow data for AMR prediction, early susceptibility forecasting, and risk stratification. Examples include ICU/EHR prediction, EHR/socioeconomic prediction, and LIS-based early susceptibility forecasting (12–14). Genomic and metagenomic studies used WGS or mNGS data for AMR prediction, resistance-gene mapping, cross-country validation, or rapid resistance inference (25–30). MALDI-TOF studies used routine diagnostic mass-spectrometry spectra for rapid AMR prediction or resistance screening (31, 32, 34, 36). Environmental and One Health studies used wastewater, sewage, environmental, food-chain, animal-sector, and farm-level data to support AMR tracking, early warning, or cross-sector surveillance (26, 37, 38, 41, 44, 45).

The second layer is harmonization and governance. Because human, animal, food-chain, environmental, and wastewater data are generated through different sampling systems and laboratory workflows, surveillance intelligence depends on standardised AMR phenotypes, interoperable metadata, data-quality control, and cross-sector linkage under appropriate privacy and bias safeguards (37, 38, 41). Privacy-preserving and federated approaches, in which models are trained across institutions and sectors by exchanging model parameters rather than patient-level or sector-level records, offer one route to cross-sector learning while protecting data confidentiality (61).

The third layer is the machine-learning and analytics engine. Across the reviewed evidence this layer applied tree-based, neural, deep-learning, MALDI-TOF spectral, anomaly-detection, Bayesian, and time-series methods. Representative examples include symbolic-regression ICU prediction, XGBoost and convolutional-network WGS-based *Salmonella* prediction, MALDI-TOF LightGBM/MLP resistance prediction, and Bayesian broiler-chain early warning (12, 26, 31, 44). Foundation models and large language models are an emerging addition to this layer; they are being tested for antibiotic decision support but are not yet validated for autonomous AMR decisions (57, 58).

The fourth layer is intelligence outputs. In the reviewed evidence, outputs included patient-level AMR risk scores, predicted susceptibility or resistance, MDRO or MRSA risk estimates, hospital or regional prevalence forecasts, rapid resistance signals from MALDI-TOF, WGS, or mNGS, wastewater or environmental resistome signals, ARG risk profiles, and animal or food-chain early-warning indicators. Some outputs are designed for bedside stewardship or infection-control decisions, while others are designed for laboratory interpretation, regional surveillance, wastewater monitoring, environmental tracking, food-chain surveillance, or One Health situational awareness. Representative examples include CRPA risk stratification, WGS-based *Salmonella* AMR prediction, wastewater surveillance, and food-animal AMR forecasting (18, 26, 41, 45).

The fifth layer links intelligence outputs to decision support and action. Clinical models may support empiric therapy selection, antimicrobial stewardship, isolation precautions, or prioritisation of high-risk patients (13, 18, 23). Genomic, MALDI-TOF, mNGS, and laboratory-based models may support earlier resistance inference and laboratory decision support (27, 29, 31, 32, 36). Environmental and wastewater models may support public-health alerts, hotspot detection, or targeted surveillance (39, 41). Animal-sector, food-chain, and integrated One Health models may support cross-sector response planning (26, 38, 44). The evidence supports technical feasibility across these domains, but it does not show that most models have been prospectively deployed into operational AMR response systems.

The sixth layer is the learning feedback loop. Surveillance intelligence should be updated using observed outcomes, resistance trends, workflow impact, model calibration, data drift, and policy or clinical response. This is necessary because AMR epidemiology, diagnostic workflows, prescribing patterns, pathogen distributions, and data-generating systems can change over time. The need for feedback and updating is supported by the broader pattern of retrospective model development and the limited number of studies with external, temporal, cross-site, or drift-focused validation. Representative validation-oriented examples include cross-country genomic validation, external EHR validation, temporal MALDI-TOF validation, and DRIAMS cross-site/temporal MALDI-TOF analyses (23, 25, 31, 36). Overall, the framework positions AMR surveillance intelligence as a learning system rather than a single prediction model.

### Comparison with existing frameworks and novelty

The proposed framework is intended to complement, not replace, established guidance for trustworthy health AI. Reporting and quality standards such as TRIPOD+AI (46) and PROBAST specify how individual prediction models should be reported and appraised, and DECIDE-AI (56) structures the early clinical evaluation of decision-support systems. Broader governance guidance, including the WHO guidance on the ethics and governance of artificial intelligence for health (62) and the FUTURE-AI consensus principles of fairness, universality, traceability, usability, robustness, and explainability (63), sets out ethical and operational conditions for deployable AI. These frameworks operate mainly at the level of a single model or a single clinical tool.

The novelty of the One Health AMR Surveillance Intelligence Framework lies in three features that these standards do not by themselves provide. First, it is specific to AMR and spans human, animal, food-chain, environmental, wastewater, and genomic data within one structure rather than addressing a single model in a single setting. Second, it positions reporting, governance, and evaluation standards as components within a larger surveillance system, linking data harmonization, analytics, intelligence outputs, decision pathways, and feedback rather than treating model appraisal as the endpoint. Third, it makes the distinction between technical prediction and actionable surveillance intelligence explicit and operational, so that existing standards such as TRIPOD+AI, DECIDE-AI, FUTURE-AI, and the WHO guidance can be applied at the appropriate layer. In this way the framework aligns with these standards and organises where each applies within an AMR One Health surveillance programme.

### Validating the framework

The framework is a conceptual synthesis, and it is presented as a testable structure rather than a validated system. A staged validation strategy would allow it to be evaluated in practice. In a first stage, retrospective application would use the framework to classify existing AMR machine-learning studies and surveillance programmes by layer and by readiness tier, testing whether the layers and the prediction-to-intelligence distinction capture the evidence without gaps or overlap. In a second stage, prospective pilot implementation in one or more settings, for example a hospital antimicrobial stewardship service, a diagnostic laboratory using MALDI-TOF or sequencing, or a wastewater surveillance programme, would instrument each layer with predefined metrics. Candidate metrics include data completeness and harmonization at the input layer, discrimination and calibration at the analytics layer, timeliness and interpretability at the output layer, change in clinician or public-health action at the decision layer, and model drift and retraining frequency at the feedback layer. In a third stage, early clinical evaluation of any decision-support component would follow DECIDE-AI (56), individual prediction models would be reported against TRIPOD+AI (46), and overall trustworthiness would be assessed against the FUTURE-AI principles (63). A deployed stewardship tool that reduced antibiotic mismatch in routine outpatient care provides a concrete precedent for the decision and feedback layers and a template for such prospective evaluation (52). Success would be defined not by model discrimination alone but by measurable and sustained improvement in surveillance and stewardship action.

### Implementation and economic considerations

Moving from a working model to an operational surveillance component carries practical and economic costs that shape feasibility. Development and training of deep-learning and genomic models can require substantial computation and specialised hardware, while routine inference for many clinical and laboratory tools, such as MALDI-TOF spectral classifiers or tree-based clinical models, is comparatively light and can run on standard hospital infrastructure. The larger and more recurrent costs usually arise after deployment. Models degrade as pathogens, prescribing, and data systems change, so periodic revalidation and retraining are needed to maintain performance, as shown by the decline in MALDI-TOF classifier accuracy over 18 months without retraining (36).

Sustained operation also depends on data engineering, laboratory and information-technology integration, interoperable metadata, quality control, clinical governance, and staff training, none of which are one-time expenses. A systematic review of economic evaluations of health-care AI found that rigorous cost and cost-effectiveness evidence remains limited, so the economic case for most tools is still unproven (64). These considerations bear directly on equity. Settings with constrained computation, sequencing capacity, connectivity, or laboratory infrastructure may be unable to sustain data-intensive tools, which risks concentrating benefit in well-resourced systems. Sustainable adoption therefore depends on matching model complexity to available infrastructure, favouring interpretable and low-maintenance approaches where appropriate, budgeting for monitoring and retraining from the outset, and reporting implementation and cost alongside accuracy. The single deployed tool in this evidence set shows that operational integration is achievable, but also that such evidence remains rare (52).

### Future directions and research priorities

Several priorities would move the field from technical feasibility toward operational surveillance intelligence. Prospective, multicentre, and cross-country validation is the most pressing need, because most current evidence is retrospective and internally validated, and external and temporal performance often falls. Multimodal approaches that combine electronic health records, laboratory, genomic, and imaging or spectral data may improve prediction where single data streams are insufficient, provided they are validated and calibrated rather than only benchmarked (15). Foundation models and large language models are entering AMR decision support, but current performance is uneven, and their use should be evaluated against dedicated reporting guidance before any autonomous role (57–59). Explainable AI should move beyond feature-attribution displays toward explanations that demonstrably support correct clinical, laboratory, or public-health decisions and that expose subgroup performance and bias, consistent with prediction-model reporting standards (46) and trustworthy-AI principles (63). Federated and privacy-preserving learning offers a route to cross-institution and cross-sector models without sharing patient-level or sector-level data, which suits One Health surveillance (61).

Finally, these methods should be connected to established public-health architecture, including alignment and interoperability with WHO surveillance and policy systems such as the Global Antimicrobial Resistance and Use Surveillance System and the Global Action Plan on Antimicrobial Resistance, so that model outputs feed coordinated One Health monitoring and response rather than remaining standalone tools (5, 7). Progress against these priorities, evaluated with prospective designs and standardised reporting, would let the field show not only that resistance can be predicted but that surveillance intelligence changes decisions and outcomes.

## Conclusion

Machine learning is now being applied to AMR across the full One Health spectrum, from clinical and electronic-health-record systems, laboratory and susceptibility workflows, and whole-genome and metagenomic sequencing to MALDI-TOF spectra, wastewater and environmental monitoring, and animal and food-chain systems. Across these settings it supports prediction, risk stratification, forecasting, rapid resistance inference, and early warning.

The decisive message of this review is that the field demonstrates technical feasibility far more convincingly than operational readiness. Most models are still developed and validated retrospectively, whereas external and prospective validation, calibration and drift monitoring, and evidence of real-world impact remain uncommon. AMR data become surveillance intelligence not when a model is merely accurate, but when its outputs are validated in the target setting, calibrated and monitored over time, interpretable, explicitly linked to a clinical, laboratory, environmental, or One Health action, and re-evaluated as epidemiology and workflows change.

Closing this gap will require standards-based development and reporting, external and prospective validation across sites and over time, explicit coupling of model outputs to decisions, and cross-sector data governance, with prediction models reported against TRIPOD+AI and PROBAST and decision-support tools evaluated early against DECIDE-AI. The proposed One Health AMR Surveillance Intelligence Framework organises this shift from data generation toward actionable, adaptive intelligence. The task for the next phase of the field is no longer to show that resistance can be predicted, but to build surveillance systems that change what clinicians, laboratories, and public-health agencies actually do.

## Data Availability

The original contributions presented in the study are included in the article/Supplementary material, further inquiries can be directed to the corresponding author.
