# Supplementary material for: From surveillance to intelligence: a scoping review of machine learning for antimicrobial resistance surveillance intelligence across One Health
Source: Front Public Health. 2026 Sep 17;14:1922265. doi: 10.3389/fpubh.2026.1922265 (PMC13627374; doi:10.3389/fpubh.2026.1922265)
Supplement: Supplementary file 1 [file Supplementary_file_1.docx]

**SUPPLEMENTARY FILE S1**

**Database Search Strategies**

Three electronic bibliographic databases were searched: PubMed/MEDLINE, Scopus, and Web of Science Core Collection. The search combined three concept blocks with the Boolean operator AND, each block built from controlled-vocabulary terms (where available) and free-text keywords combined with OR.

Date range: 1 January 2015 to 1 May 2026. Language: English. All databases were last searched on 1 May 2026. Database searching was supplemented by backward and forward citation chasing and source verification.

**Concept blocks**

Block 1: Antimicrobial resistance: antimicrobial/antibiotic/drug resistance, multidrug-resistant organisms, resistance genes, resistome, antimicrobial susceptibility.

Block 2: Machine learning / data-analytic methods: machine learning, artificial intelligence, deep learning, neural networks, predictive/prediction models, forecasting, anomaly detection, MALDI-TOF spectral modelling, language models.

Block 3: Surveillance-intelligence functions: surveillance, tracking, risk stratification, risk prediction, early warning, decision support, hotspot/outbreak monitoring, One Health.

**PubMed / MEDLINE**

#1 ("Drug Resistance, Bacterial"[Mesh] OR "Drug Resistance, Microbial"[Mesh] OR

"Drug Resistance, Multiple, Bacterial"[Mesh] OR "Microbial Sensitivity Tests"[Mesh] OR

"antimicrobial resistance"[tiab] OR "antibiotic resistance"[tiab] OR "drug resistance"[tiab] OR

"multidrug-resistant"[tiab] OR "multidrug resistant"[tiab] OR MDRO[tiab] OR "resistance gene*"[tiab] OR

resistome[tiab] OR "antimicrobial susceptibility"[tiab] OR AMR[tiab])

#2 ("Machine Learning"[Mesh] OR "Artificial Intelligence"[Mesh] OR "Deep Learning"[Mesh] OR

"Neural Networks, Computer"[Mesh] OR "Supervised Machine Learning"[Mesh] OR "Forecasting"[Mesh] OR

"machine learning"[tiab] OR "artificial intelligence"[tiab] OR "deep learning"[tiab] OR

"neural network*"[tiab] OR "random forest"[tiab] OR XGBoost[tiab] OR "gradient boosting"[tiab] OR

"support vector machine*"[tiab] OR "predictive model*"[tiab] OR "prediction model*"[tiab] OR

forecasting[tiab] OR "anomaly detection"[tiab] OR "language model*"[tiab] OR "MALDI-TOF"[tiab])

#3 ("Epidemiological Monitoring"[Mesh] OR "Public Health Surveillance"[Mesh] OR

"Population Surveillance"[Mesh] OR "One Health"[Mesh] OR surveillance[tiab] OR tracking[tiab] OR

"early warning"[tiab] OR "risk stratification"[tiab] OR "risk prediction"[tiab] OR

"decision support"[tiab] OR "One Health"[tiab] OR hotspot*[tiab] OR outbreak*[tiab] OR monitoring[tiab])

#4 #1 AND #2 AND #3

Limits: ("2015/01/01"[Date - Publication] : "2026/05/01"[Date - Publication]) AND English[Language]

**Scopus**

( TITLE-ABS-KEY ( "antimicrobial resistance" OR "antibiotic resistance" OR "drug resistance" OR

"multidrug-resistant" OR MDRO OR "resistance gene*" OR resistome OR "antimicrobial susceptibility" OR AMR )

AND TITLE-ABS-KEY ( "machine learning" OR "artificial intelligence" OR "deep learning" OR

"neural network*" OR "random forest" OR XGBoost OR "gradient boosting" OR "support vector machine*" OR

"predictive model*" OR "prediction model*" OR forecasting OR "anomaly detection" OR "language model*" OR

"MALDI-TOF" )

AND TITLE-ABS-KEY ( surveillance OR tracking OR "early warning" OR "risk stratification" OR

"risk prediction" OR "decision support" OR "One Health" OR hotspot* OR outbreak* OR monitoring ) )

AND PUBYEAR > 2014 AND PUBYEAR < 2026

AND ( LIMIT-TO ( LANGUAGE , "English" ) )

**Web of Science**

TS=("antimicrobial resistance" OR "antibiotic resistance" OR "drug resistance" OR "multidrug-resistant"

OR MDRO OR "resistance gene*" OR resistome OR "antimicrobial susceptibility" OR AMR)

AND

TS=("machine learning" OR "artificial intelligence" OR "deep learning" OR "neural network*" OR

"random forest" OR XGBoost OR "gradient boosting" OR "support vector machine*" OR "predictive model*"

OR "prediction model*" OR forecasting OR "anomaly detection" OR "language model*" OR "MALDI-TOF")

AND

TS=(surveillance OR tracking OR "early warning" OR "risk stratification" OR "risk prediction" OR

"decision support" OR "One Health" OR hotspot* OR outbreak* OR monitoring)

Refined by: Publication Years = 2015-2026; Languages = English. Date last searched: 1 May 2026.
