# Supplementary material for: From surveillance to intelligence: a scoping review of machine learning for antimicrobial resistance surveillance intelligence across One Health
Source: Front Public Health. 2026 Sep 17;14:1922265. doi: 10.3389/fpubh.2026.1922265 (PMC13627374; doi:10.3389/fpubh.2026.1922265)
Supplement: Supplementary file 2 [file Supplementary_file_2.docx]

**PRISMA-ScR CHECKLIST**

| **Section** | **Item** | **PRISMA-ScR checklist item** | **Reported in (location / page)** |
| --- | --- | --- | --- |
| **TITLE** | | | |
| Title | 1 | Identify the report as a scoping review. | Title page (states “scoping review”). p. 01 |
| **ABSTRACT** | | | |
| Structured summary | 2 | Structured summary: background, objectives, eligibility, sources, charting methods, results, conclusions. | Abstract (Background/Objective/Methods/Results/Conclusions). p. 01 |
| **INTRODUCTION** | | | |
| Rationale | 3 | Describe the rationale in the context of what is already known. | Introduction. p. 02 |
| Objectives | 4 | Provide an explicit statement of the questions and objectives. | Introduction; Methods — Review question. p. 04 |
| **METHODS** | | | |
| Protocol and registration | 5 | Indicate whether a protocol exists; state registration information. | Methods — Study design (OSF registration). p. 04 |
| Eligibility criteria | 6 | Specify characteristics of sources of evidence used as eligibility criteria. | Methods — Eligibility criteria (Population–Concept–Context). p.04__ |
| Information sources | 7 | Describe all information sources and date last searched. | Methods — Information sources and search strategy (last searched 1 May 2026). p. 05 |
| Search | 8 | Present the full electronic search strategy for at least one database. | Methods (summary) and Supplementary File S1 (full strategies, all 3 databases). p. 05 |
| Selection of sources of evidence | 9 | State the process for selecting sources (i.e., screening and eligibility). | Methods — Record management and study selection; two reviewers, independent. p. 04 |
| Data charting process | 10 | Describe the methods of charting data (independently; in duplicate; any process for confirming data). | Methods — Data extraction (charted by two reviewers; piloted form). p. 05 |
| Data items | 11 | List and define all variables for which data were sought and charted. | Methods — Data extraction (charted variables list). p. 05 |
| Critical appraisal (optional) | 12 | If done, describe methods of critical appraisal of sources of evidence. | Methods — Quality, validation, and implementation appraisal (TRIPOD+AI/PROBAST-aligned). p. 05 |
| Synthesis of results | 13 | Describe the methods of handling and summarizing the charted data. | Methods — Evidence synthesis (structured narrative synthesis). p. 05 |
| **RESULTS** | | | |
| Selection of sources of evidence | 14 | Give numbers screened, assessed for eligibility, and included, with reasons for exclusions (flow diagram). | Results — Study selection; PRISMA-ScR flow diagram (Figure 1). p. 06 |
| Characteristics of sources of evidence | 15 | For each source, present characteristics for which data were charted. | Results; Tables. p. 08-10 |
| Critical appraisal within sources (optional) | 16 | If done, present critical appraisal data for each source. | Results; Table 6, Figure 5 and per-study appraisal. p. 33 |
| Results of individual sources of evidence | 17 | For each source, present the relevant data that relate to the review questions/objectives. | Tables 1–5 (per-study). p. 08-22 |
| Synthesis of results | 18 | Summarize and/or present the charting results as they relate to the review questions/objectives. | Results narrative; Figures 1,3,4, 5 p.22, 33 |
| **DISCUSSION** | | | |
| Summary of evidence | 19 | Summarize main results (including overview of concepts); relate to review questions; consider relevance to key groups. | Discussion — Principal findings. p. 28 |
| Limitations | 20 | Discuss limitations of the scoping review process. | Discussion — Strengths and limitations (incl. language restriction). p. 35 |
| Conclusions | 21 | Provide a general interpretation of the results with respect to the questions and objectives; note implications and/or next steps. | Conclusion. p. 37 |
| **FUNDING** | | | |
| Funding | 22 | Describe sources of funding and the role of funders for the scoping review. | Funding statement - . p. 38 |
